# Effect of Buffers with Multiple Binding Sites on Calcium Waves

**DOI:** 10.1007/s11538-022-01109-0

**Published:** 2022-12-31

**Authors:** Bogdan Kazmierczak, James Sneyd, Je-Chiang Tsai

**Affiliations:** 1 Institute of Fundamental Technological Research, Polish Academy of Sciences, Pawinskiego 5B, 02-106 Warsaw, Poland; 2 Department of Mathematics, University of Auckland, 38 Princes St, Auckland, 1010 New Zealand; 3 Department of Mathematics, National Tsing Hua University, No. 101, Sec. 2, Kuang-Fu Road, Hsinchu, 300 Taiwan; 4 National Center for Theoretical Sciences, No.1, Sec. 4, Roosevelt Road, Taipei, 106 Taiwan

**Keywords:** Reaction-diffusion systems, Buffered calcium systems

## Abstract

The existence and properties of intracellular waves of increased free cytoplasmic calcium concentration (calcium waves) are strongly affected by the binding and unbinding of calcium ions to a multitude of different buffers in the cell. These buffers can be mobile or immobile and, in general, have multiple binding sites that are not independent. Previous theoretical studies have focused on the case when each buffer molecule binds a single calcium ion. In this study, we analyze how calcium waves are affected by calcium buffers with two non-independent binding sites, and show that the interactions between the calcium binding sites can result in the emergence of new behaviors. In particular, for certain combinations of kinetic parameters, the profiles of buffer molecules with one calcium ion bound can be non-monotone.

## Introduction

The spatio-temporal distribution of calcium ions plays a significant role in the control of cellular processes such as fertilization, proliferation, morphogenetic development, gene expression, learning and memory, synaptic communication, muscle contraction, hormone secretion, cell movement and wound repair. In particular, in many cell types a wide range of stimuli (typically mediated by the binding of hormones or neurotransmitters to receptors on the cell surface) initiate oscillations and waves of increased calcium concentration, and it is the dynamic properties of these oscillations and waves (in particular the period, localization, and amplitude) which act as the intracellular signal (Dupont et al. 2016; Falcke 2004–2005).

Although the processes that control the cytoplasmic calcium concentration differ in detail between cell types, there is considerable overlap between the basic mechanisms, and thus it makes sense to talk of a generic calcium oscillation or wave model. Such a generic model relies on the excitable nature of the release of calcium from the endoplasmic reticulum (ER), whereby a small increase in cytoplasmic calcium concentration can lead to the release of much greater amounts of calcium from the ER, typically either through inositol trisphosphate receptors (e.g., in non-excitable cells), through ryanodine receptors (e.g., in striated muscle cells), or through both types of channels. Such excitable release of calcium from internal stores is generically called calcium-induced calcium release, or CICR (Dupont et al. 2016).

Because of the inherently excitable nature of CICR, an understanding of the dynamical behavior of calcium waves can be gained by the analytical study of the well-known FitzHugh–Nagumo (FHN) excitable model (Fitzhugh 1960, 1961; Nagumo et al. 1962). Further, in many cell types calcium release happens on a much faster time scale than the recovery process, and thus the FHN model can be reduced to the single bistable model. This reduction is equivalent to the study of the leading front of wave solutions in the FHN model.

However, the mechanism for controlling calcium waves differs from the traditional excitable mechanism in one crucial respect: the presence of large numbers of calcium buffer proteins. In normal conditions, more than 99% of cytoplasmic calcium ions are bound by buffer proteins. This is because free calcium ions are poisonous to the cell (because they activate so many things), and so buffers are used to control the concentration of free calcium. Therefore, in order to study calcium waves, it is necessary to investigate the dynamical behavior of buffered excitable systems. Along this line, numerous researchers have studied calcium wave propagation in buffered excitable systems with the presence of calcium buffers with a single binding site. The buffered bistable model was proposed by Sneyd et al. (1998). The existence and uniqueness of waves are established by a number of authors (Tsai and Sneyd 2005; Kaźmierczak and Volpert 2008a, b), and stability is shown in Tsai (2007). One needs to note that not every wave in mathematical sense is physiologically relevant. By this we mean that the elevated stable equilibrium of calcium concentration should expand into the region of lower stable equilibrium (ground state). A criterion for the existence of a physiologically relevant wave is derived in Tsai and Sneyd (2011, Proposition 3.2).

Previous work on the effects of calcium buffers on calcium dynamics has mostly assumed that calcium buffering can be modeled effectively by assuming that each buffer molecule has a single calcium binding site. This assumption is, in general, not satisfied; calcium buffers typically bind multiple calcium ions in a cooperative fashion, with the binding of one calcium ion affecting the rate at which calcium binds to the other binding sites (Schwaller 2010). However, recent work by Matveev and his colleagues (Matveev 2018; Chen and Matveev 2021) has extended the traditional analysis of buffers with a single binding site to include the effects of a second binding site, where the binding is cooperative. It is this general approach that we follow here.

It is important to note that cooperative binding is critical for our analysis. If the buffer molecules have several independent calcium binding sites we can represent the action of these buffers as a sum of buffer molecules each with a single binding site (see e.g., Sect. 6 in Kaźmierczak and Sneyd 2021) . However, the assumption of cooperative binding means that the binding sites are not independent, and the probability of calcium binding to any particular site will depend on which other binding sites are already occupied.

In particular, we consider the extreme situation in which the calcium ions can bind only consecutively, concentrating mainly on the case of two binding sites. Thus initially, calcium can bind only to the first site and the other sites are unavailable (hidden). After binding a calcium ion to this site, the second consecutive site is activated, e.g., exposed as a result of changing the buffer molecule conformation. This process can repeat consecutively. Unbinding of calcium follows an analogous process.

Another important fact is that due to the change of conformation, the buffer molecule can have a different diffusion coefficient than initially (Sorensen and Shea 1996), which additionally complicates the description. This phenomenon will be, however, not taken into account.

In this paper, we show that buffered systems with buffer molecules possessing multiple dependent calcium binding sites can have specific properties, not shared by the systems with buffer molecules having only one binding site (or independent binding sites).

The plan of this paper is as follows. In Sect. 2, we present the mathematical model. Section 3 is devoted to traveling wave problem and its fast buffering reduction. The validity of the fast buffering reduction is given in the appendix. Then, in Sect. 4, a criterion for the existence of a physiologically acceptable wave is derived. With this criterion, the effect of two-site buffers on the dynamical behavior of calcium waves is deduced in Sect. 5. Finally, conclusions and discussions are given in Sect. 6.

## Buffered Bistable Model

Let us consider the case of buffer molecules which can bind $m \ge 2$ calcium ions. For simplicity, we will confine to one single type of buffer molecules. Now set

$$\begin{aligned} M_j = \mathrm{[M}_j], \quad j=0,\ldots ,m, \end{aligned}$$

where $\textrm{M}_j$ represents the buffer molecule which binds exactly *j* calcium ions. Note that $M_0$ stands for the unbound form of buffers. Let the process of binding and unbinding of a calcium ion to the buffer molecule $\textrm{M}_j$ be described by the following reaction scheme with the kinetic constants $k^{j}_{+}$ and $k^{j}_{-}$, respectively:

1 $$\begin{aligned} \textrm{Ca}^{2+}+\textrm{M}_{j}\;\; \mathop {\rightleftarrows }_{k^{j}_{-}}^{k^{j}_{+}}\;\; \textrm{Ca}^{2+}\textrm{M}_{j} \equiv \textrm{M}_{j+1},\;\; j=0,\ldots ,m-1. \end{aligned}$$

Let *c* and $c_b$ denote the concentration of free cytosolic calcium and the basal concentration of free cytosolic calcium, respectively. Let *D* denote the diffusion coefficient of free calcium ions, and $D_{M_j} \ge 0$, $j=0,\ldots ,m$, the diffusion coefficient of the *j*-th buffer $M_j$ with calcium bound ($D_{M_j}=0$ means that the *j*-th buffer is immobile). Assuming, for simplicity, that the cell is one-dimensional, and using the law of mass-action, we obtain the *Buffered bistable* model:

2 $$\begin{aligned} c_t= & {} D_c c_{xx} + f(c) + \underbrace{\sum _{j=0}^{m-1} \left[ - k_{+}^{j} c M_{j} + k_{-}^{j} M_{j+1} \right] }_{\text{ calcium } \text{ buffering }}, \nonumber \\ M_{j,t}= & {} D_{M_j} M_{j,xx} + \left[ k_{+}^{j-1} c M_{j-1} - k_{-}^{j-1} M_{j} - k_{+}^{j} c M_{j}+ k_{-}^{j} M_{j+1} \right] , \nonumber \\{} & {} \quad \quad j=1, \ldots , m-1, \nonumber \\ M_{m,t}= & {} D_{M_m} M_{m,xx} + \left[ k_{+}^{m-1} c M_{m-1} - k_{-}^{m - 1} M_{m} \right] , \nonumber \\ M_{0,t}= & {} D_{M_0} M_{0,xx} - \left[ k_+^0 M_0 c - k_-^0 M_1 \right] . \end{aligned}$$

here *f*(*c*) denotes the calcium fluxes in and out of the cytoplasm, and, in general, it contains a number of terms such as release from inositol trisphosphate receptors, reuptake by pumps, and so on. Since the free cytosolic calcium possesses one high stable steady state of calcium concentration in the models for calcium waves, we use the bistable nonlinearity for *f*. Specifically, *f* is assumed to take the well-known bistable nonlinearity

3 $$\begin{aligned} f(c)= & {} {{\mathcal {S}}}\big (c-c_1\big ) \big (c-c_2\big ) \big ( c_3-c\big ) \nonumber \\:= & {} {{\mathcal {S}}} \big (c-c_b\big ) \big (c-(a+c_b)\big ) \big ( (1+c_b)-c\big ) \end{aligned}$$

where ${{\mathcal {S}}} > 0$ and $a\in (0,1)$ are constants. The zeros of *f* have the following biological implications (see Smith et al. 2002 for more details): (i) the state $c_1$ represents a stable resting state at basal calcium concentration in cytosol; (ii) the state $c_3$ is a state at high calcium concentration in the cytosol; and (iii) the state $c_2$ corresponds to a threshold for the activation process (e.g., CICR).

According to what we said in Introduction, we do not take into account a possible difference in diffusion coefficient due to change of buffer molecules conformation. Therefore, throughout this paper we assume that

$$\begin{aligned} D_{M_0}= D_{M_1}= \ldots =D_{M_m}:=D_M. \end{aligned}$$

Then, by adding the equations for $M_j$, $j=0,1, ..., m,$ we conclude that

$$\begin{aligned} \left( \sum _{j=0,\dots ,m} M_j \right) _{,t} = D_M\left( \sum _{j=0,\dots ,m} M_j \right) _{,xx}. \end{aligned}$$

The only solution of this equation which is bounded uniformly on $\mathrm{I\!R}$ is identically a constant. We thus have

$$\begin{aligned} \sum _{j=0,\dots ,m} M_j = b_0, \end{aligned}$$

where $b_0$ represents the constant which is the total concentration of buffer molecules at each spatial point. Consequently, we can replace $M_0$ by $b_0-\sum _{j=1,\dots ,m} M_j$ and consider the resulting system consisting of the first $m+1$ equations of system (2). Then, system (2) is reduced to the following system

4 $$\begin{aligned} c_t{} & {} = D_c c_{xx} + f(c) - k_+^0 b_0 c\nonumber \\{} & {} \quad + \sum _{j=1}^{m-1} \left[ \left( k_+^0 - k_+^j \right) cM_j + k_-^{j-1}M_j \right] + \left( k_+^0 c + k_-^{m-1} \right) M_m, \nonumber \\ M_{1,t}{} & {} = D_{M} M_{1,xx} \nonumber \\{} & {} \quad + \, k^0_+ b_0 c - \left( k^0_- + k^0_+ c + k^1_{+} c \right) M_1 + \left( k^1_{-} -k^0_+ c \right) M_2 - \sum _{j=3}^m k^0_+ c M_j, \nonumber \\ M_{j,t}{} & {} = D_{M_j} M_{j,xx}\nonumber \\{} & {} \quad + \left[ k_{+}^{j-1} c M_{j-1} - k_{-}^{j-1} M_{j} - k_{+}^{j} c M_{j}+ k_{-}^{j} M_{j+1} \right] , \quad j=2, \ldots , m-1, \nonumber \\ M_{m,t}{} & {} = D_{M_m} M_{m,xx} + \left[ k_{+}^{m-1} c M_{m-1} - k_{-}^{m - 1} M_{m} \right] . \end{aligned}$$

It is seen that the equations for $M _j$, $j=2,\ldots ,m$, do not change, and that the equation for $M_1$ is modified. We note that for $m \ge 3$ the obtained system is not monotone, so does not enjoy the comparison principle and the existence theorem of traveling waves in Volpert et al. (1994) due to the form of the equation for $M_1$.

## Traveling Wave Problem and Fast Buffering Reduction

### Buffered Bistable Model with Two Calcium-Binding Sites

To facilitate the discussion for the effect of multiple binding sites on traveling waves, we will only focus on the case where a single type of buffer is present, and such a type of buffer possesses exactly two calcium binding sites. Under the aforementioned assumption, system (4) is reduced to the following *Buffered bistable system*:

5 $$\begin{aligned} c_t= & {} D_c c_{xx} + f(c) - \left[ k^0_+ b_0 c + \left( k^1_{+} c - k^0_{+} c - k^0_{-} \right) M_1 - \left( k^0_{+} c + k^1_{-} \right) M_2 \right] , \nonumber \\ M_{1,t}= & {} D_{M} M_{1,xx} + k^0_+ b_0 c - \left( k^0_- + k^0_+ c + k^1_{+} c \right) M_1 + \left( k^1_{-} -k^0_+ c \right) M_2, \nonumber \\ M_{2,t}= & {} D_{M} M_{2,xx} + \left[ k^1_{+} M_1 c - k^1_- M_2 \right] . \end{aligned}$$

A direct computation reveals that the constant states of system (5) are given by the following expressions:

6 $$\begin{aligned} P_{j} := \left( c_j,M^j_1,M^j_2 \right) , \quad j=1,2,3, \end{aligned}$$

where the $c_j$ are defined in (3), and the $M^j_1$ and $M^j_2$ are given by

7 $$\begin{aligned} M^j_1 = \dfrac{k^0_+ b_0 c_j}{k^0_- + k^0_+ c_j + k^0_+ \dfrac{k^1_{+}}{k^1_-} c_j^2} , \quad M^j_2= \dfrac{k^1_{+}}{k^1_-} c_j M^j_1. \end{aligned}$$

### Traveling Wave Problem for Two-site Buffering Model

A traveling wave solution $(c, M_1, M_2)$ of system (5) connecting $P_1$ to $P_3$ is a solution of system (5) which is a function of the traveling wave coordinate variable $\xi =x+vt$, i.e.

8 $$\begin{aligned} (c, M_1, M_2)(x,t)=(c, M_1, M_2)(\xi ), \end{aligned}$$

and satisfies the boundary conditions

9 $$\begin{aligned} \lim _{\xi \rightarrow -\infty }(c, M_1, M_2)(\xi )=P_1 \;\text { and }\; \lim _{\xi \rightarrow +\infty }(c, M_1, M_2)(\xi )=P_3, \end{aligned}$$

where *v* is the wave speed.

We make one remark about the notation of the traveling wave coordinate. Since we use $\xi =x+vt$ as the traveling wave coordinate, a wave solution with positive (resp. negative) wave speed *v* corresponds to a wave propagating from the right to the left (resp. from the left to the right).

Note that not all of wave solutions are biologically reasonable. When waves pass throughout the whole cytosol, the free cytosolic calcium is at a high stable steady state. Therefore, a positive wave speed *v* is required for a biologically reasonable wave solution.

In terms of the moving coordinate $\xi =x+vt$, the wave profile $(c, M_1, M_2)$ of a traveling wave solution of system (5) satisfies the following ordinary differential equations:

10 $$\begin{aligned} \begin{array}{l} D_c c'' - v c' + f(c) - \left[ k^0_+ b_0 c + \left( k^1_{+} c - k^0_{+} c - k^0_{-} \right) M_1 - \left( k^0_{+} c + k^1_{-} \right) M_2 \right] = 0, \\ D_{M} M''_1 - v M'_1 + k^0_+ b_0 c - \left( k^0_- + k^0_+ c + k^1_{+} c \right) M_1 + \left( k^1_{-} -k^0_+ c \right) M_2 = 0, \\ D_{M} M''_2 - v M'_2 + \left[ k^1_{+} M_1 c - k^1_- M_2 \right] = 0. \end{array} \end{aligned}$$

subject to the boundary condition (9).

### Rapid Buffering Model

In the following, we will apply the *rapid buffering approximation* (RBA) (Wagner and Keizer 1994; Keener and Sneyd 1998) to analyze traveling wave solutions to system (5). The RBA was first proposed by Wagner and Keizer (1994) to study the effect of rapid buffers with a single binding site on calcium diffusion and oscillations. Its central idea is to assume that buffering processes are very fast compared with the other reactions. Mathematically, this means that

$$\begin{aligned} k^j_\pm \gg 1 \;\, \text {with} ~\frac{k^j_-}{k^j_+}~\text { being constant}, \,\, j = 0, 1. \end{aligned}$$

According to the RBA, we will use the second and the third equations in system (10) (the equation for $M_1$) to calculate the expression $I_b$ in the bracket of the first equation of system (10), which in turn reduce the full system to a single equation for the free calcium concentration. Here we give an outline of this approach. To proceed, the expression $I_b$ can be rewritten in the following form:

11 $$\begin{aligned} I_b:= & {} \left[ k^0_+ b_0 c + \left( k^1_{+} c - k^0_{+} c - k^0_{-} \right) M_1 - \left( k^0_{+} c + k^1_{-} \right) M_2 \right] \nonumber \\= & {} \left[ k^0_+ b_0 c - \left( k^0_- + k^0_+ c + k^1_{+} c \right) M_1 + \left( k^1_{-} -k^0_+ c \right) M_2 \right] \nonumber \\{} & {} + \, 2 \left[ k^1_{+} M_1 c - k^1_- M_2 \right] \nonumber \\=: & {} I_{b1} + 2 I_{b2} \end{aligned}$$

where $I_{b1}$ and $I_{b2}$ denote the free terms in the second and third equation of system (10). The presence of factor 2 in the last equality of (11) follows from the fact that every $M_2$ molecule has two calcium ions bound. We will thus calculate $I_{b1}$ and $I_{b2}$, which in turn gives the value of $I_b$.

Next, we rescale the expressions for $I_{b1}$ and $I_{b2}$ using the large parameter out of them. The rescaled expressions are then equated to zero, to obtain the dependence of the functions $M_1$ and $M_2$ on the variable *c*. Finally, by differentiation we calculate the $D_M M''_1 -v M'_1$ and $D_M M''_2 -v M'_2$, and thus obtain the values of the unrescaled quantities $I_{b1}$ and $I_{b2}$, and so the $I_b$.

Now, we carry out the RBA for system (10). The rigorous verification of this procedure is postponed to the appendix. As a large parameter *L* we take any of the quantities $k^{0,1}_\pm$, e.g., $L = k^0_+$. Let us set

12 $$\begin{aligned} k^{0,1}_\pm = : L \kappa ^{0,1}_\pm . \end{aligned}$$

Then, system (10) can be written as:

13 $$\begin{aligned} \begin{array}{l} D_c c'' - v c' + f(c) - L \left[ \kappa ^0_+ b_0 c + \left( \kappa ^1_{+} c - \kappa ^0_{+} c - \kappa ^0_{-} \right) M_1 - \left( \kappa ^0_{+} c + \kappa ^1_{-} \right) M_2 \right] = 0 \\ D_{M} M''_1 - v M'_1 + L \left[ \kappa ^0_+ b_0 c - \left( \kappa ^0_- + \kappa ^0_+ c + \kappa ^1_{+} c \right) M_1 + \left( \kappa ^1_{-} -\kappa ^0_+ c \right) M_2 \right] = 0 \\ D_{M} M''_2 - v M'_2 + L \left[ \kappa ^1_{+} M_1 c - \kappa ^1_- M_2 \right] = 0 \end{array} \end{aligned}$$

As the coefficients $\kappa$ are of the order of 1, so we can write $I_{b1}$ and $I_{b2}$ in the form:

14 $$\begin{aligned} \begin{array}{l} I_{b1} = L \left[ \kappa ^0_+ b_0 c - \left( \kappa ^0_- + \kappa ^0_+ c + \kappa ^1_{+} c \right) M_1 + \left( \kappa ^1_{-} -\kappa ^0_+ c \right) M_2 \right] = :L I_{1}, \\ I_{b2} = L \left[ \kappa ^1_{+} M_1 c - \kappa ^1_- M_2 \right] =:L I_{2}. \end{array} \end{aligned}$$

The quantities $I_1$ and $I_2$ do not depend on any large parameters, so, asymptotically, we are justified to demand $I_1=0$ and $I_2=0$. From the second equation we obtain

15 $$\begin{aligned} M_2 =\dfrac{\kappa ^1_{+}}{\kappa ^1_-} M_1 c. \end{aligned}$$

Putting this relation into $I_1$ and equating it to zero, we obtain

16 $$\begin{aligned} M_1(c) = \dfrac{\kappa ^0_+ b_0 c}{\kappa ^0_- + \kappa ^0_+ c + \kappa ^0_+ \dfrac{\kappa ^1_{+}}{\kappa ^1_-} c^2} =\dfrac{ b_0 c}{{{\mathcal {K}}}(c) } \end{aligned}$$

with $K_0$, $K_1$ and ${{\mathcal {K}}}$ defined by

17 $$\begin{aligned} K_{0} := \dfrac{k^0_-}{k^0_+}=\dfrac{\kappa ^0_-}{\kappa ^0_+}, \quad K_1:= \dfrac{k^1_-}{k^1_+} = \dfrac{\kappa ^1_-}{\kappa ^1_+}, \quad \mathcal{K}(c):=K_0 + c + K_1^{-1}c^2. \end{aligned}$$

Now differentiating (16) gives

18 $$\begin{aligned} \dfrac{d M_1}{dc}(c) = : \theta _1(c) = \dfrac{b_0 (K_0 - K_1^{-1} c^2)}{{{\mathcal {K}}}(c)^2}. \end{aligned}$$

Hence, in the moving coordinate $\xi = x + vt$, we have

$$\begin{aligned} M'_1(\xi ) = \theta _1(c(\xi )) \,c'(\xi ). \end{aligned}$$

Now, a further differentiation of $M_1'(\xi )$ with respect to $\xi$ gives that

19 $$\begin{aligned} -I_{b1}= & {} D_M M_1'' - v M_1' \nonumber \\= & {} D_M \big [ \theta _1'(c(\xi )) (c'(\xi ))^2 + \theta _1(c(\xi )) c''(\xi ) \big ] - v \theta _1(c(\xi )) c'(\xi ). \end{aligned}$$

Likewise,

20 $$\begin{aligned} M_2(c) = K_1^{-1} M_1(c) c = K_1^{-1} c \dfrac{ b_0 c}{{{\mathcal {K}}}(c)} \end{aligned}$$

and

21 $$\begin{aligned} \dfrac{d M_2}{dc}(c) =:\theta _2(c) = K_1^{-1} \dfrac{b_0 c (c+2 K_0)}{\mathcal{K}(c)^2} \end{aligned}$$

with

$$\begin{aligned} M'_2(\xi ) = \theta _2(c(\xi )) \,c'(\xi ). \end{aligned}$$

Hence

22 $$\begin{aligned} -I_{b2}= & {} D_M M_2'' - v M_2' \nonumber \\= & {} D_M \big [ \theta _2'(c(\xi )) (c'(\xi ))^2 + \theta _2(c(\xi )) c''(\xi ) \big ] - v \theta _2(c(\xi )) c'(\xi ). \end{aligned}$$

Finally, by plugging (11), (19), and (22) into the *c*-equation of system (13), it follows that the *c*-equation of system (13), and so that of system (10), can be rewritten as the following *Rapid buffered bistable* system:

23 $$\begin{aligned} \left( D_c + D_M \theta (c) \right) c'' +2 D_M \theta '(c) \, (c')^2 - v (1+\theta (c)) c' + f(c) = 0, \end{aligned}$$

where

24 $$\begin{aligned} \theta (c)= & {} \theta _1(c) + 2 \theta _2(c) = \dfrac{b_0 \left( K_1^{-1} c^2 + 4 c K_0 K _1^{-1}+ K_0 \right) }{{{\mathcal {K}}}(c)^2} \nonumber \\= & {} \dfrac{b_0 \left( K_1^{-1} c^2 + 4 c K_0 K _1^{-1}+ K_0 \right) }{ \left( K_0 + c + K_1^{-1}c^2 \right) ^2} \end{aligned}$$

It is worthwhile to note that Eq. (23) corresponds to the parabolic equation of the form:

25 $$\begin{aligned} (1+\theta (c)) c_t = \left( D_c + D_M \theta (c) \right) c_{xx} +2 D_M \theta '(c) \, (c_{x})^2 + f(c) \end{aligned}$$

under the traveling wave ansatz $c(x,t) = c(\xi ) = c(x+vt)$.

Now, under the assumption of buffers with fast kinetics, a traveling wave solution of the Buffering system (5) can be approximated by a solution of the Rapid buffering system (23), as stated in the following proposition.

#### Proposition 1

(Rapid buffering reduction) Let scaling (12) be in force. Suppose that $(v_0, c_0)$ is a solution of the Rapid buffered bistable system (23) subject to the boundary conditions

$$\begin{aligned} \lim _{\xi \rightarrow -\infty } c_0(\xi ) = c_b \;\text { and }\; \lim _{\xi \rightarrow +\infty } c_0(\xi ) = 1 + c_b. \end{aligned}$$

Then, there exists a large $L > 0$ such that for each $L > L_0$, we can find a traveling wave solution $(v_L,c_L,M_{1,L},M_{2,L})$ of the Buffering bistable system (5) such that

$$\begin{aligned}{} & {} |v_L - v_0| = O(L^{-1}),\quad \Vert c_L - c_0\Vert _{C^2(\mathrm{I\!R})} = O(L^{-1}), \\{} & {} \Vert M_{1,L} - M_1(c_0)\Vert _{C^2(\mathrm{I\!R})} = O(L^{-1}), \quad \Vert M_{2,L} - M_2(c_0)\Vert _{C^2(\mathrm{I\!R})} = O(L^{-1}), \end{aligned}$$

where $M_1(\cdot )$ and $M_2(\cdot )$ are defined by (16) and (20), respectively.

The validity of Proposition 1 will be shown in the Appendix. (See the proof of a more general Theorem A.1.)

### A Useful Transformation $\phi$

Let us note that given the function $\theta$, the structure of Eq. (23) is the same as the structure of Eq. (12.34) in Keener and Sneyd (1998). Let us note that Eq. (23) can be written as

26 $$\begin{aligned} \left( w(c) \right) '' - v (1+\theta (c)) c' + f(c) = 0, \end{aligned}$$

where

27 $$\begin{aligned} w(c):= D_c c + D_M \left( M_1(c) + 2 M_2(c) \right) = D_c c + D_M b_0 c \,\, \dfrac{ 1 + 2 K_1^{-1} c }{{{\mathcal {K}}}(c)} \, . \end{aligned}$$

Now, as $w'(c) = D_c + D_M \theta (c)$, then *w* is a monotonically increasing function of *c*. So, *w* and *c* are inverse functions of each other. Let us denote

28 $$\begin{aligned} c = c(w) =:\phi (w). \end{aligned}$$

We have

29 $$\begin{aligned} \dfrac{\hbox {d} \phi }{\hbox {d} w} = \left( \dfrac{\hbox {d} w}{\hbox {d}c} \right) ^{-1} = \dfrac{1}{D_c + D_M \theta (c(w)) }, \end{aligned}$$

thus

30 $$\begin{aligned} \frac{\hbox {d} \phi }{\hbox {d}w} (w) >0 \quad \text{ for } ~w \in \mathrm{I\!R}_{+} \cup \{0\}. \end{aligned}$$

Equation (26) can thus be converted into an equation for *w*:

31 $$\begin{aligned} w'' - v (1+\theta (c(w))) \dfrac{1}{D_c + D_M \theta (c(w)) } w' + f(\phi (w)) = 0. \end{aligned}$$

## A Criterion for the Propagation of Buffered Waves

### Theorem 1

(Existence and uniqueness of wave solutions of Rapid buffering system)

Let $w_1$ and $w_3$ be the unique real numbers such that $\phi (w_1) = c_b$ and $\phi (w_3)=1+c_b$.

Suppose that

32 $$\begin{aligned} \int _{w_1}^{w_3} f\big (\phi (w) \big ) \hbox {d}w > 0. \end{aligned}$$

Then, there exist a positive increasing function $c_0:\mathrm{I\!R}\rightarrow \mathrm{I\!R}$ and a positive number $v_0$ such that $(v_0, c_0)$ is a solution of the Rapid buffering system (23)–(24) subject to the boundary conditions

$$\begin{aligned} \lim _{\xi \rightarrow -\infty } c_0(\xi ) = c_b \;\text { and }\; \lim _{\xi \rightarrow +\infty } c_0(\xi ) = 1 + c_b. \end{aligned}$$

Moreover, the solution pair $(v_0,c_0)$ is unique in the sense that if $(v_0^*,c_0^*)$ is another solution pair, then $v_0=v_0^*$ and $c_0=c_0^*(\cdot +\xi _0)$ on $\mathrm{I\!R}$ for some $\xi _0\in \mathrm{I\!R}$.

The proof of the theorem uses the standard shooting method and the arguments from Sneyd et al. (1998) applied to the case of buffers with one binding site, but for completeness and better reference below, we insert it here.

### Proof of Theorem 1

According to the analysis given in Sect. 3.4, it suffices to consider Eq. (31). To begin with, we will give local analysis of (31) around its singular points. To see this, we can write (31) as a first-order system:

33 $$\begin{aligned} \begin{array}{l} w' =z \\ z' = \Psi (w) \, v \,z - f(\phi (w)) \end{array} \end{aligned}$$

where

$$\begin{aligned} \Theta (w):= & {} \theta (\phi (w)) = \dfrac{b_0 \left( K_1^{-1} \phi (w)^2 + 4 \phi (w) K_0 K_1^{-1}+ K_0 \right) }{{{\mathcal {K}}}(\phi (w))^2}, \\ \Psi (w):= & {} \dfrac{1+\Theta (w)}{D_c + D_M \Theta (w)}. \end{aligned}$$

The steady states of this system are equal to $(w_i,0)$, $i=1,2,3$, where

34 $$\begin{aligned} \phi (w_1)=c_b, \quad \phi (w_2) = c_b + a, \quad \phi (w_3) = c_b+1. \end{aligned}$$

Noting that $\phi$ and *w* are inverse functions of each other by (27) and (28), this implies

35 $$\begin{aligned} w_1=w(c_b), \quad w_2 = w(c_b + a), \quad w_3 = w(c_b+1) \end{aligned}$$

Due to the fact that

$$\begin{aligned} \dfrac{\hbox {d} f(\phi (w))}{\hbox {d} w} = \dfrac{\hbox {d} f(\phi (w))}{\hbox {d} \phi (w)} \cdot \dfrac{\hbox {d} \phi }{\hbox {d}w} \end{aligned}$$

and using (30) and (34), we conclude that the states $w_1$ and $w_3$ are stable, whereas $w_2$ is unstable. In fact, we have

$$\begin{aligned} \begin{array}{l} \dfrac{\hbox {d} f(\phi (w) )}{\hbox {d} w} \Big |_{w=w_1} = -{{\mathcal {S}}}a \dfrac{1}{D_c + D_M \theta (c_b) } =: d_1<0 \, , \\ \dfrac{\hbox {d} f(\phi (w) )}{\hbox {d} w} \Big |_{w=w_2} = {{\mathcal {S}}}a(1-a) \dfrac{1}{D_c + D_M \theta (a+c_b) } =: d_2 >0 \, , \\ \dfrac{\hbox {d} f(\phi (w) )}{\hbox {d} w} \Big |_{w=w_3} = -{{\mathcal {S}}}(1-a) \dfrac{1}{D_c + D_M \theta (1+c_b) } =: d_3 <0 \,. \end{array} \end{aligned}$$

The eigenvalues of the linearization matrices at the singular points $(w_i,0)$, $i=1,2,3$, are solutions to the equations

$$\begin{aligned} \lambda _i^2 - \lambda \, v \Psi (w_i) + d_i = 0 \end{aligned}$$

and are given by the expressions:

$$\begin{aligned} \lambda _{i \pm } = \dfrac{1}{2} \left( v \Psi (w_i) \pm \sqrt{ v^2 \Psi ^2(w_i) - 4 d_i} \right) . \end{aligned}$$

It follows that $(w_2,0)$ is a repeller, whereas $(w_i,0)$, $i=1,2$ are saddle points. The eigenvectors corresponding to the positive eigenvalue at $(w_1,0)$ and to the negative eigenvalue at $(w_3,0)$ can be written as:

36 $$\begin{aligned} \begin{array}{l} V_1 = \left( 1, \dfrac{2 d_1}{ v \Psi (w_1) - \sqrt{v^2 \Psi ^2(w_1) - d_1}} \right) =: (1,S_1(d_1,v)), \\ V_3 = \left( -1, \dfrac{-2 d_3}{ v \Psi (w_3) + \sqrt{v^2 \Psi ^2(w_3) - d_3}} \right) =: (-1,S_3(d_3,v)). \end{array} \end{aligned}$$

Before proceeding further, we make two observations. First, as $d_1<0$, $d_3<0$, then for $v \ge 0$ the vector $V_1$ has a positive slope, while the vector $V_2$ has a negative slope. Moreover, given $d_1<0$, the function $S_1$ is a strictly increasing function of *v* with $S_1(d_1,0)=2 \sqrt{|d_1|}$ and $\lim _{v \rightarrow \infty } S_1(d_1,v)=\infty$. On the other hand, the function $S_3$ decreases with $v \ge 0$. Next, if $z(\xi )=w'(\xi )>0$ for $\xi$ in some interval $(\xi _1,\xi _2)$, then *z* can be treated also as a function of *w* via the identification $z(w) = z(w(\xi ))=z(\xi )$. Below, for simplicity, we will use the same symbol for *z* as a function of *w*.

Now, we go to the shooting scheme. To proceed, let us note that, for given $v \ge 0$, the trajectories of system (33 ) starting from $(w_1,0)$ along the vector $V_1$ satisfy the equation

37 $$\begin{aligned} \dfrac{1}{2} z^2(w) = v \int _{w_1}^w \Psi (w) z(w) dw - \int _{w_1}^w f\big (\phi (w) \big ) dw. \end{aligned}$$

Due to the form of $f(\cdot )$, we have $f(\phi (w) )<0$ for $w \in (w_1,w_2)$, and hence

38 $$\begin{aligned} \text{ for }~ v \ge 0~, ~z(w)> 0~ \text {and}~ z_{,w}(w)>0~\text { for}~{ w \in (w_1,w_2].} \end{aligned}$$

Now, we consider the trajectory with $v=0$. Indeed, from (38) the trajectory with $v=0$ will lie above the *w*-axis for $w \in (w_1,w_2]$. On the other hand, according to (37) and (32), for $v=0$ the trajectory must touch the axis $z=0$ for some $w<w_3$. Taken together, it follows that for $v=0$, the trajectory must intersect the axis $z=0$ for some $w_0 \in (w_2,w_3)$.

Next, we consider the trajectory with sufficiently large *v*. To proceed, let us first consider the case $v>0$. Then, due to the fact that $f(\phi (w) )<0$ for $w \in (w_1,w_2)$, we have by (37):

$$\begin{aligned} z^2(w_2)> -2 \int _{w_1}^{w_2} f\big (\phi (w) \big ) dw =: z^2_0. \end{aligned}$$

Since system (33) is autonomous, without losing generality, we can suppose that, given $v>0$, $w_2=w(\xi )|_{\xi =0}$. Thus, by the *z*-equation of (33), we conclude that, as long as $w(\xi ) \le w_3$, $z(\xi )=w'(\xi )$ satisfies the inequality

$$\begin{aligned} z'(\xi )> v {\underline{\Psi }} z - {\overline{f}}, \quad z(\xi =0)>z_0, \end{aligned}$$

where ${\underline{\Psi }} := \inf _{w \in (w_2,w_3)} \Psi (w)$ and ${\overline{f}} := \sup _{w \in (w_2,w_3)} f(\phi (w) )$. Set ${\tilde{z}}:=z- {\overline{f}}/(v {\underline{\Psi }})$. Then, the last inequality implies that for $\xi >0$

$$\begin{aligned} {\tilde{z}}'>v {\underline{\Psi }} {\tilde{z}}, \quad {\tilde{z}}(0)=z(0) - \frac{{\overline{f}}}{v {\underline{\Psi }}} > z_0 - \frac{{\overline{f}}}{v {\underline{\Psi }}}. \end{aligned}$$

It follows that, if $v>0$ is sufficiently large, then ${\tilde{z}}(0) > z_0/2$, and so for all $\xi >0$

$$\begin{aligned} z(\xi )> \dfrac{z_0}{2} \exp (v {\underline{\Psi }} \xi ) + \frac{{\overline{f}}}{v {\underline{\Psi }}} > \dfrac{z_0}{2}. \end{aligned}$$

As a result, given sufficiently large $v>0$, the considered trajectory will cross the line $w=w_3$ for some $z = z_v > z_0/2$. The boundedness of $z_v$ is implied by the fact that for any solution *z* such that $z(w)>0$ and $z_{,w}(w)>0$ for all $w \in (w_1,w_3]$, we have

$$\begin{aligned} z^2(w_3) = v \int _{w_1}^{w_3} z(w) \Psi (w) \hbox {d}w - \int _{w_1}^{w_3} f\big (\phi (w) \big ) \hbox {d}w < v z(w_3) \int _{w_1}^{w_3} \Psi (w) \hbox {d}w, \end{aligned}$$

where we used (32). This leads to the estimate

$$\begin{aligned} z(w_3) < v \int _{w_1}^{w_3} \Psi (w) \hbox {d}w. \end{aligned}$$

To summarize, for $v=0$ the trajectory of system (33) crosses the axis $z=0$ for $w \in (w_2,w_3)$, and there exists $v^*>0$ sufficiently large such that the corresponding trajectory crosses the line $w=w_3$ for some finite $z_{v^*}>0$. By using the continuity argument, we conclude that there must exist at least one $v_0 \in (0,v^*)$, such that for $v=v_0$ the corresponding trajectory reaches the singular point $(w_3,0)$. Moreover, such a $v_0$ is unique. Suppose to the contrary that there exist $v_{01}$ and $v_{02}>v_{01}$, for which the corresponding trajectories $T_1$ and $T_2$ join the points $(w_1,0)$ and $(w_3,0)$. Then, the trajectory $T_2$ starts at a bigger slope than $T_1$, and, by the monotonicity of trajectories with respect to $v \ge 0$, $T_2$ stays above $T_1$ for all $w \in (w_1,w_3)$. However, according to the first observation after (36) $T_2$ should cross $T_1$ from above, hence we arrive at contradiction. This completes the proof. $\square$

## Separatrix Curve $J_C=0$ and Buffers’ Effect

As it is seen from Theorem 1 and its proof, the ’sine qua non’ condition for the existence of traveling waves with positive speed is the positivity of the integral

39 $$\begin{aligned} \int _{w_1}^{w_3} f\big (\phi (w) \big ) \hbox {d}w = \int _{c_b}^{1+c_b} \left( D_c + D_M \theta (c)) \right) f(c) \hbox {d}c. \end{aligned}$$

However, as the value of the integral $\int _{c_b}^{1+c_b} D_c f(c)dc$ is known, similarly to Tsai (2013), we will consider only the integral

40 $$\begin{aligned} J_C(a,K_0,K_1){} & {} := b_0^{-1} \int _{c_b}^{1+c_b} \theta (c) f(c) \hbox {d}c\nonumber \\{} & {} = \int _{c_b}^{1+c_b} f(c) \, \dfrac{ \left( K_1^{-1} c^2 + 4 c K_0 K _1^{-1}+ K_0 \right) }{ \left( K_0 + c + K_1^{-1} c^2 \right) ^2}\hbox {d}c. \end{aligned}$$

We note that if $J_C(a,K_0,K_1) > 0$, then calcium traveling waves always propagate. On the other hand, if $J_C(a,K_0,K_1) < 0$, then waves will cease to propagate if the product $D_M b_0$ is large enough so that

$$\begin{aligned} \int _{c_b}^{1+c_b} D_c f(c) \hbox {d}c + D_M b_0 J_C(a,K_0,K_1) < 0. \end{aligned}$$

Therefore, the presence of buffers can prevent the propagation of calcium waves only if their kinetic characteristics satisfy $J_C(a,K_0,K_1) < 0$. This suggest a detailed study of the separatrix curve $J_C(a,K_0,K_1) = 0$, which will be done later.

Before proceeding, let us note that, according to (1) and (17), the limit $K_1 \rightarrow \infty$ corresponds to the case of buffers with single calcium binding site. Biologically, this can be explained by the fact that $K_1 = \frac{k_-^1}{k_+^1}$, so $K_1$ tending to infinity means that the reaction

$$\begin{aligned} \textrm{Ca}^{2+}+\textrm{M}_{1}\;\; \mathop {\rightleftarrows }_{k^{1}_{-}}^{k^{1}_{+}}\;\; \textrm{M}_{2}. \end{aligned}$$

runs only from the right to the left. Therefore, in fact there are no molecules with two calcium ions bound. Also note that in the limit $K_1 \rightarrow \infty$, the right-hand side of (40) is reduced to

41 $$\begin{aligned} J_C(a,K_0) = \int _{c_b}^{1+c_b} f(c) \left[ \dfrac{K_0}{(c+K_0)^2} \right] \hbox {d}c. \end{aligned}$$

Therefore, the condition that

42 $$\begin{aligned} J_C(a,K_0) < 0. \end{aligned}$$

is a necessary condition enabling one-site buffers to slow down or stop the propagation of advancing calcium waves.

### The Modified System Excitability Function $a(K_0,K_1)$

As we have noted, the separatrix curve $J_C(a,K_0,K_1) =0$ plays a key role in the propagation of calcium traveling waves. In this subsection, we will investigate it in more detail. To proceed, we will use the equation $J_C(a,K_0,K_1) =0$ to deduce that the excitability variable *a* can be represented as a function $a(\cdot , \cdot )$ of the kinetic characteristic pair $(K_0, K_1)$. Let us remind that according to (3) we have assumed the source function has the cubic form

43 $$\begin{aligned} f(c)=f(a;c)= {{\mathcal {S}}}(c-c_b)(c-c_b-a)(1-(c-c_b)) \end{aligned}$$

where $a \in (0,1)$ and ${{\mathcal {S}}}$ is a positive constant. Recall that the parameter *a* in the source function *f* characterizes the system excitability. Therefore, the function $a(K_0, K_1)$ characterizes the system excitability in the buffered system with the kinetic characteristic pair $(K_0, K_1)$. Further, for a given buffered system with the kinetic characteristic pair $(K_0, K_1)$, if $0< a < a(K_0, K_1)$, then $J_C(a,K_0,K_1) > 0$, and so waves always propagate. On the other hand, if $a > a(K_0, K_1)$, then $J_C(a,K_0,K_1) < 0$, and so waves may cease to propagate provided if the product ($b_0 D_M$) of the concentration of total buffers and their diffusion coefficients are large enough. These follow from the fact that the function $J_C(a,K_0,K_1)$ is decreasing in *a*, as shown in the following lemma.

#### Lemma 1

For all $a \in [0,1]$, $K_0 \in [0,\infty )$ and $K_1 \in [0,\infty )$, we have

$$\begin{aligned} \dfrac{\partial J_C}{\partial a}(a,K_0,K_1) < 0. \end{aligned}$$

#### Proof

The proof follows from the fact that due to (43) for $c \in (c_b,c_b+1)$

$$\begin{aligned} \dfrac{\hbox {d} f}{\hbox {d} a}(a;c) = -{{\mathcal {S}}} (c - c_b) \cdot (1 - (c - c_b)) < 0. \end{aligned}$$

$$\square $$

With the use of Lemma 1, the separatrix curve $J_C(a,K_0,K_1) =0$ gives rise to the existence of the modified excitability function $a(\cdot , \cdot )$, as shown in the following lemma.

#### Lemma 2

(Existence of the modified system excitability function $a(K_0,K_1)$) There exists a function $a:[0,\infty ) \times [0,\infty ) \mapsto [0,\infty )$ such that, for all $K_0 \in [0,\infty )$ and $K_1 \in [0,\infty )$, we have

$$\begin{aligned} J_C \left( a(K_0,K_1),K_0,K_1 \right) = 0. \end{aligned}$$

For all $\epsilon _1>0$, $\epsilon _2>0$, the function $a(\cdot ,\cdot )$ is of $C^1$ class of their arguments on the set $[\epsilon _1,\infty ) \times (\epsilon _2,\infty )$.

#### Proof

The existence of the function $a(\cdot , \cdot )$ follows from the implicit function theorem and Lemma 1. Likewise, the differentiability follows from the fact that, for $j=0,1$, we have

44 $$\begin{aligned} \dfrac{\partial a}{\partial K_j}(K_0,K_1) = - \, { \dfrac{ \partial J_C}{\partial K_j} (a,K_0,K_1)} \, \Big ( {\dfrac{\partial J_C}{\partial a} (a,K_0,K_1)} \Big )^{-1}, \end{aligned}$$

from which (and Theorem 1) follows the boundedness and continuity of the derivatives. $\square$

It should be noted that for $(K_0,K_1)$ tending to $(0, \infty )$

$$\begin{aligned} J_{C,a} (a,K_0,K_1) \rightarrow K_0 \int _{c_b}^{1+c_b} f_a(a;c) c^{\!-\!2} dc \!+\! K_1^{\!-\!1} \int _{c_b}^{1+c_b} f_a(a;c) dc \!+\! h.o.t. \rightarrow 0. \end{aligned}$$

Next, $J_{C}(a,0,K_1) \rightarrow K_1^{-1} \int _{c_b}^{1+c_b} f(a;c) dc +O(K_1^{-2})$, from where it follows that $a(0,K_1) = 0.5 - |O(K_1^{-1})|$. Now, $J_{C,K_0}(a,0,K_1) \rightarrow \int _{c_b}^{1+c_b} f(a;c) c^{-2} dc + O(K_1^{-2}) = \int _{c_b}^{1+c_b} f(0.5;c) c^{-2} dc + O(K_1^{-1})<0$. It follows, according to (44), that $a_{K_0} (0,K_1) \rightarrow \infty$ as $K_1 \rightarrow \infty$. These properties of the function $a(K_0,K_1)$ are illustrated in the right panel of Fig. 2. Similar singularity analysis can be carried out for $(K_0,K_1) \rightarrow (\infty ,0)$.

**Fig. 1:**
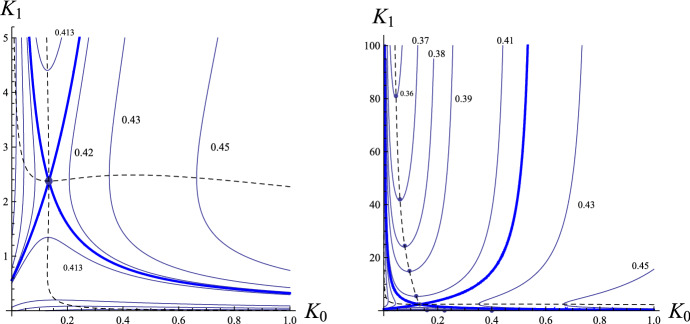
Form of separatrix curves $J_C(a,K_0,K_1)=0$ for $c_b=0.2$, for different values of $a \in (0,1)$ and for $K_0 \in [0,1]$. Left panel: The case for small range of $K_1 \in [0,5]$. The dashed almost vertical curve corresponds to the set of points for which $a(K_0,K_1)_{,K_0} = 0$, whereas the dashed almost horizontal curve corresponds to the set of points for which $a(K_0,K_1)_{,K_1} = 0$. The bold curves correspond to the two branches of the curve associated with the curve $J_C(a^*,K_0,K_1)=0$, where the critical parameter $a^* \approx 0.4174$. These branches meet together at the critical point $(K_0^*,K_1^*)$ of the modified excitability function $a(K_0, K_1)$. The three lowest curves are depicted in the smaller scale in the left panel of Fig. 2. The three curves in the left-hand side sector correspond (from right to left) to $a=0.42, 0.43$ and 0.45. Right panel: The case for large range of $K_1 \in [0,100]$. The curves have the same meanings as those in the left panel

**Fig. 2:**
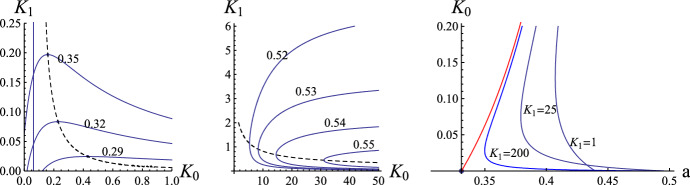
Left panel: The three lowest curves $J_C(a,K_0,K_1)=0$ in the left panel graph of Fig. 1 corresponding to $a=0.35, \, 0.32$ and 0.29. The vertical straightline demarcates from the right the values of $K_0$ for which $J_C(0.35,K_0,K_1) \le 0$ in the case of buffer molecules with only one site of calcium binding. The dashed curve corresponds to the set of points for which $a(K_0,K_1)_{,K0} = 0$ with $a(K_0,K_1)_{,K0} >0$ to the right and $a(K_0,K_1)_{,K0} \le 0$ to the left of it. Middle panel: Form of separatrix curves $J_C(a,K_0,K_1)=0$ for large values of $K_0 \in [0,1]$. Right panel: Shape of the curves $a(K_0,K_1)$ for fixed chosen values of $K_1$. As $K_1 \rightarrow \infty$, $a_{K_0}(0,K_1) \rightarrow \infty$

Though the exact expressions of the modified excitability function $a(\cdot , \cdot )$ can be obtained (e.g., by using *Mathematica* code), they very complicated, thus it is better to use numerical simulations for the analysis. To fix our attention, in the remainder of this section, we will choose

45 $$\begin{aligned} c_b = 0.2 \, \, (\mu M) \end{aligned}$$

for the calcium ground concentration, unless it is indicated differently. The resting cytoplasmic calcium concentration is typically around 100 nM, but can vary anywhere from 20 nM to over 200 nM (Clapham 2007). The value we choose here is thus slightly on the high side, but still within the physiological regime.

For the case of one-site buffers, the exact expression of the function $a(\cdot , \cdot )$ is given by Sneyd et al. (1998). For two-site buffers, approximate (asymptotic) formulae can be only derived in some specific cases. For example, this can be done for very large values of $K_0$, as it is shown in Sect. 5.2.2.

The shapes of the curves $J_C(a,K_1,K_1) = 0$ for some values of $a \in (0, 1)$ are depicted in Fig. 1. The critical point $(K_0^*, K_1^*) \approx (0.133, 2.376)$ of the modified excitability function $a(\cdot , \cdot )$ for which $\partial a/\partial K_0(K_0^*, K_1^*) = 0$ and $\partial a/\partial K_1(K_0^*, K_1^*) = 0$ divides the family of the curves $J_C(a,K_1,K_1) = 0$ into two classes. Specifically, let $a^* = a(K_0^*, K_1^*) \approx 0.417425$ be the critical excitability parameter. Then, for $a \in (0, a^*)$, the curve $J_C(a,K_1,K_1) = 0$ consists of two parts: one is opening upwards, and the other is opening downwards. On the other hand, for $a \in (a^*, 1)$, one component of the curve $J_C(a,K_1,K_1) = 0$ opens to the left, and the other opens to the right. This can be seen from the left and right panels of Fig. 1. The presentation of the form of the curves $J_C(a,K_1,K_1) = 0$ is continued in Fig. 2. In the left panel of Fig. 2 we show the region of smaller values of $K_1$, whereas in the middle panel the region with larger values of $K_0$. Finally, in the right panel we show the form of the curves $a(K_0;K_1)$ as a function of $K_0$ for some given values of $K_1$.

### The Effect of Two-site Buffers

In this subsection, we analyze of the effect of binding sites on traveling wave solutions.

#### Admissible Region $\mathcal {A}$ for Wave Propagation

In this subsection, we discuss the region in the $K_0K_1$ plane for which the corresponding wave propagates from the right to the left, hence with $v>0$. If these conditions are satisfied we will simply say, for brevity, that the waves propagate.

To proceed, recall that for a fixed $a \in (0,1)$, if the kinetic pair $(K_0, K_1)$ lies in the region

$$\begin{aligned} \mathcal {A} := \big \{ (K_0, K_1) \in \mathrm{I\!R}^2_+: \, J_C(a,K_0,K_1) > 0 \big \}, \end{aligned}$$

then the corresponding wave always propagates. On the other hand, if $(K_0, K_1)$ falls outside the region $\mathcal {A}(K_0, K_1)$, then waves cannot propagate provided if the product $b_0 D_M$ is large enough.

In order to be compared with one-site buffers, we need to define a critical $K_0$. Indeed, for a given $a \in (0, 1/2)$, there exists a critical $K_0^s \ge 0$ such that

$$\begin{aligned} J_C(a, K_0) < 0 \,\text { for }\, K_0 \in (0, K_0^s) \,\text { and }\, J_C(a, K_0)> 0 \,\text { for }\, K_0 > K_0^s. \end{aligned}$$

**Fig. 3:**
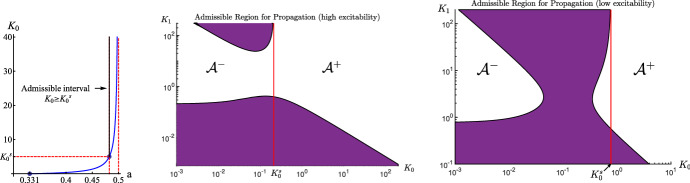
Admissible region $\mathcal {A} = \mathcal {A}^+\bigcup \mathcal {A}^-$ in the $K_0K_1$ plane for which waves can propagate for $c_b=0.2$. Left panel: Admissible region of propagation for one-site buffer molecules. Middle panel: $a=0.38$. $K_0^s =0.2104$. Right panel: $a=0.43$. $K_0^s= 0.7736$ . The one-site buffered wave can propagate if $K_0 \ge K_0^s$ (independently of the value $D_M b_0$)

Therefore, for the case of one-site buffers, when $K_0 > K_0^s$, waves always propagate, whereas for $K_0 \in (0, K_0^s)$, waves will be stopped provided if the product $b_0 D_M$ is large enough. As it is seen from the left panel of Fig. 3, the minimal excitability parameter *a* for which the action of buffer molecules can stop the advancing wave propagation is approximately equal to 0.331. On the other hand, for two site buffers, it can be shown that the minimal value of *a* is approximately not bigger than 0.254. (For example, for $K_0=10$ and $K_1=0.00007$ it is equal to 0.254.)

These differences are also expressed in the regions in the $K_0K_1$-space, in for which the advancing waves can propagate independently of the values of $D_M b_0$.

With the use of $K_0^s$, the region $\mathcal {A}$ is decomposed into two subregions $\mathcal {A}^+$ and $\mathcal {A}^-$, defined by

$$\begin{aligned} \mathcal {A}^+= & {} \mathcal {A} \bigcap \big \{ (K_0, K_1) : \, K_0 \ge K_0^s, K_1> 0 \big \}, \\ \mathcal {A}^-= & {} \mathcal {A} \bigcap \big \{ (K_0, K_1) : \, K_0 \in (0, K_0^s), K_1 > 0 \big \}. \end{aligned}$$

The regions $\mathcal {A}$, $\mathcal {A}^+$, $\mathcal {A}^-$, for two characteristic values of the parameter *a*, are depicted in Fig. 3. As shown in Fig. 3, one of the boundaries of the region $\mathcal {A}^-$ tends to the vertical line $K_0 = K_0^s$ as $K_1 \nearrow \infty$. This reflects the fact that $J_C(a, K_0, K_1) \rightarrow J_C(a, K_0)$ as $K_1 \rightarrow \infty$.

We first discuss the case where $(K_0, K_1) \in \mathcal {A}^+$. For one-site buffers, the corresponding wave always propagates. For two-site buffers, although the corresponding wave always propagates if $K_1$ is large enough. On the other hand, the wave can fail to propagate provided $K_1$ is small and the product $b_0 D_M$ is large enough. As shown in Fig. 3, such a region (bottom portion of purple color with $K_0 \ge K_0^s$) is relatively small. (Note that the graphs in the middle and right panel are in the logarithmic scale.) Hence, we can come to an approximate conclusion that two-site buffers retain propagation for the kinetic pair $(K_0, K_1) \in \mathcal {A}^+$ for which one-site buffered waves always propagate.

Next we consider the case where $(K_0, K_1) \in \mathcal {A}^-$. For one-site buffers, the corresponding wave can be stopped provided $b_0 D_M$ is large enough. However, for two-site buffers, some of the corresponding waves can propagate. Therefore, we can conclude that two-site buffers can promote propagation for the kinetic pair $(K_0, K_1) \in \mathcal {A}^-$ for which one-site buffered waves can be stopped if the product $b_0 D_M$ is large enough.

#### Can Buffers with Multiple Binding Sites Facilitate Calcium Wave Propagation?

One can ask an alternative question: Can the presence of buffers facilitate the propagation of calcium waves? Let us consider the extreme case where waves do not exist in the system with the absence of buffers, that is,

46 $$\begin{aligned} \int _{c_b}^{c_b+1} f(c) \hbox {d}c < 0. \end{aligned}$$

Then, our question is the following. Suppose that condition (46) holds. Can the addition of buffers promote the propagation of waves? Intuitively, this seems to be impossible. However, as we will see, one-site buffers cannot promote the propagation, whereas two-site buffers can empower the propagation of advancing waves.

**Single binding site**

For the case of one-site buffers, the answer is negative, as it follows from the form of the integral $J_C(a,K_0)$ given by (41) and representing (up to a positive factor) the influence of buffers. Indeed, using the mean-value theorem for integrals and the fact that *f* is one sign on the intervals $(c_b, c_b+a)$ and $(c_b+a, c_b+1)$, we can find $c_- \in (c_b, c_b+a)$ and $c_+ \in (c_b+a, c_b+1)$ such that

$$\begin{aligned} J_C(a,K_0)= & {} \int _{c_b}^{a+c_b} f(c) \left[ \dfrac{K_0}{(c+K_0)^2} \right] \hbox {d}c + \int _{a+c_b}^{1+c_b} f(c) \left[ \dfrac{K_0}{(c+K_0)^2} \right] \hbox {d}c \\[1ex]= & {} \dfrac{K_0}{(c_- + K_0)^2} \int _{c_b}^{a+c_b} f(c) \hbox {d}c + \dfrac{K_0}{(c_+ + K_0)^2} \int _{a+c_b}^{1+c_b} f(c) \hbox {d}c \\[1ex]< & {} \dfrac{K_0}{(c_- + K_0)^2} \int _{c_b}^{a+c_b} f(c) \hbox {d}c + \dfrac{K_0}{(c_- + K_0)^2} \int _{a+c_b}^{1+c_b} f(c) \hbox {d}c \\[1ex]= & {} \dfrac{K_0}{(c_- + K_0)^2} \int _{c_b}^{1+c_b} f(c) \hbox {d}c < 0. \end{aligned}$$

This in turn implies that

$$\begin{aligned} \int _{w_1}^{w_3} f\big (\phi (w) \big ) \hbox {d}w = D_c \int _{c_b}^{1+c_b} f(c)\hbox {d}c + D_M b_0 J_C(a,K_0) < 0. \end{aligned}$$

Hence, in this case the waves cannot exist by Theorem 1.

**Fig. 4:**
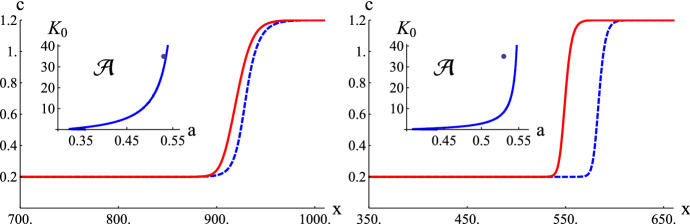
Time evolution of free calcium concentration with $a=0.53$, $K_0=35$ and $c_b=0.2$. Left panel: $K_1=0.1$. The red continuous curve corresponds to traveling wave solution of Eq. (25) generated by the initial condition $c(x,0)=0.2+H(x-1000)$ for $t=400$. The blue dashed curves correspond to traveling waves generated by system (5) by the same jump condition for *c* and the corresponding initial conditions for $M_1$ and $M_2$ for $t=400$. Parameters used in the simulations: $D=300$, $b_0=D_M=150$, $L = 50$, $\kappa ^0_{+}=\kappa ^1_{+}=1$, $\kappa ^0_{-}=35$, $\kappa ^1_{-}=0.1$, ${{\mathcal {S}}}=500$. Right panel: $K_1=1$. $L=100$, $\kappa ^1_{+}=1$. Other parameters as in the right panel. The inset figures illustrate that the corresponding parameter pair $(a, K_0)$ lies above the separatrix curve $J_C(a, K_0, K_1) = 0$ (Color figure online)

**Two Binding Sites**

For the case of two-site buffers, a successful propagation depends on whether the quantity

$$\begin{aligned} \int _{w_1}^{w_3} f\big (\phi (w) \big ) \hbox {d}w = D_c \int _{c_b}^{1+c_b} f(c)\hbox {d}c + D_M b_0 J_C(a,K_0,K_1) \end{aligned}$$

is positive or not, as indicated by Theorem 1. Since the integral $\int _{c_b}^{1+c_b} f(c)dc$ is negative, we need to look for a triplet $(a, K_0, K_1)$ such that $J_C(a,K_0,K_1) > 0$. Then, for such a triplet $(a, K_0, K_1)$, the corresponding wave will propagate provided the product $D_M b_0$ is large enough.

First, the right-hand side of (40) can be written in the form

47 $$\begin{aligned} J_C(a,K_0,K_1) := (K_0)^{-1} \cdot {\tilde{J}}_C(a,K_0,K_1) \end{aligned}$$

where for *f* given by (43),

$$\begin{aligned} \lim _{K_0 \rightarrow \infty } {\tilde{J}}_C(a,K_0,K_1)= & {} \int _{c_b}^{1+c_b} f(c) \, \left( 1+ \dfrac{4 c}{K_1} \right) \hbox {d}c \\= & {} \dfrac{12 + 20 c_b + 5 K_1 - 10 a \, (2 + 4 c_b + K_1)}{60 K_1}. \end{aligned}$$

It follows that for $K_1>0$, in the limit $K_0 \rightarrow \infty$,

48 $$\begin{aligned} a(\infty ,K_1) = \dfrac{12 + 20 c_b + 5 K_1}{10 (2 + 4 c_b + K_1)}= \dfrac{1}{2} + \dfrac{2}{10 (2 + 4 c_b + K_1)}. \end{aligned}$$

For $c_b=0.2$ and $K_1 \rightarrow 0$, $a(\infty ,K_1) \rightarrow 0.571$. We have thus shown the following theorem.

##### Theorem 2

For every fixed $K_1>0$, there exists $K_0>0$ sufficiently large, such that $a(K_0,K_1) > 1/2$. It follows that for $a<1/2$ traveling waves to Eq. (25) have positive speed ($v>0$) independently of the value of the product $D_M b_0$, whereas for all $a \in [1/2,a(K_0,K_1))$ the waves have positive speed ($v>0$) if only the product $D_M b_0$ is sufficiently large.

It must be however remarked that, according to (47), for fixed $a \in (0,1)$ and $K_1>0$, the value of the integral $J_C(a,K_0,K_1)$ decreases approximately as $(K_0)^{-1}$.

For the excitability parameters $a \in (0,1/2)$ the statement of the theorem is obvious as the limit $K_0 \rightarrow \infty$ corresponds to the situation where calcium ions are completely released from bound buffers by the reaction scheme (1) and the relation $K_0 = k_-^0/k_+^0$.

On the other hand, for $a>1/2$ the claim of the theorem seems paradoxical and it is hard to find its physical explanation. Theorem 2 is confirmed numerically. Their results are presented in Fig. 4. It is numerically shown that for $K_1=1$ (left panel) and $K_1=0.1$ (right panel), $a(K_0,K_1)>0.53$. For these parameters and $a=0.53$, there exist traveling wave solutions with $v>0$. Moreover, this result holds not only for traveling wave solutions to the asymptotic Eq. (25) (heteroclinic solutions to Eq. (23), but also to traveling waves of an initial system (5) representing Eq. (23) with sufficiently large *L*.

#### Monotonicity Versus Non-monotonicity of Wave Profiles

As the initial system of reaction diffusion Eq. (5) is in general a non-monotonic one, one can expect that the profiles of some of its components can be also non-monotone. The same remark refers to the profiles of *c*, $M_1$ and $M_2$ defined by system (23), (18) and (21). First, let us note that the profile of the *c*-component of the traveling wave solutions to Eq. (23) is always monotone increasing, as can be seen from Lemma 1 and its proof. More precisely, we have

49 $$\begin{aligned} c'(\xi ) > 0 \,\text { and }\, c_b< c(\xi ) < c_b + 1 \,\text {for all }~{\xi \in \mathrm{I\!R}.} \end{aligned}$$

Therefore, it remains to consider the profiles $M_1$ and $M_2$ of bound buffers.

**Single Binding Site**

This case corresponds to $K_1 = \infty$, hence from (18) and the fact that the *c*-component of wave solutions is monotone increasing, it follows that the $M_1$-component of wave solutions is monotone increasing. Therefore, for the case of one-site buffers, the profiles of the concentrations of free calcium ions and free/bound buffers of wave solutions are always monotonic.

**Two Binding Sites**

For the case of two-site buffers, the situation is more complicated. Let us consider the profile of the $M_1$-component of wave solutions. For the $M_1$-component of wave solutions to be increasing, by (49), we have that $M_{1,c}(c(\xi )) = M_{1,c} \, c'(\xi ) > 0$ on $\mathrm{I\!R}$. With the aid of (18), this is equivalent to the inequality $K_0 - K_1^{-1} c^2 > 0$ for $c \in (c_b, c_b+1)$, and hence that $K_0 > K_1^{-1} (c_b+1)^2$. Therefore by (18), we can conclude that

$$\begin{aligned} \dfrac{\hbox {d} M_1}{\hbox {d} \xi }>0~ \text { on}~ \mathrm{I\!R}~\,\Leftrightarrow \, K_0 > K_1^{-1} (c_b+1)^2. \end{aligned}$$

Similar arguments give that

$$\begin{aligned} \dfrac{\hbox {d} M_1}{\hbox {d} \xi }<0~ \text { on}~ \mathrm{I\!R}\,\Leftrightarrow \, K_0 < K_1^{-1} c_b^2. \end{aligned}$$

For each $K_1 > 0$ fixed, the above two observations motivate us to define the following two curves in the parameter $(a, K_0)$-plane:

$$\begin{aligned} \Gamma _l{} & {} := \{(a, K_0):\, K_0 - K_1^{-1} c_b^2=0\},\\{} & {} \text {(the solid (lowest) horizontal line in Fig.}~5 \text {and}~ 6 )~ \\ \Gamma _h{} & {} := \{(a, K_0):\, K_0 - K_1^{-1} (1+c_b)^2=0\}.\\{} & {} \text {(the dotted (highest) horizontal line in Fig.}~5 ~\text {and}~ 6) \end{aligned}$$

**Fig. 5:**
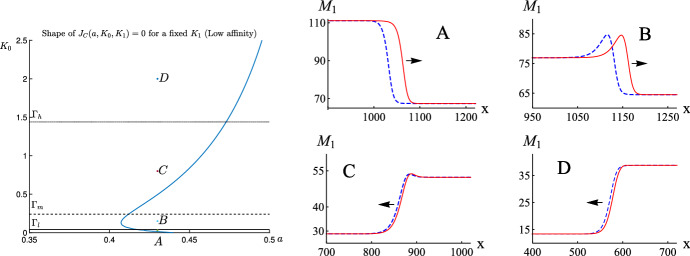
Left panel: The shape of the separatrix curve $J_C(a, K_0, K_1)=0$ for $c_b=0.2$ and a fixed $K_1=1$ in the $aK_0$ plane. Right panel: The profiles of the $M_1$ component of wave solutions corresponding to the following parameter pairs $(a, K_0)$: $A=(0.43,0.03)$, $B=(0.43,0.15)$, $C=(0.43,0.8)$, $D=(0.43,2)$ shown in the left panel. The red continuous curves correspond to traveling wave solutions of Eq. (25) generated by the initial condition $c(x,0)=0.2+H(x-1000)$ for $t=200$. The blue dashed curves correspond to traveling waves generated by system (5) by the same jump condition for *c* and the corresponding initial conditions for $M_1$ and $M_2$. Parameters used in the simulations: $D=300$, $L = 15$, $\kappa ^0_{+}=\kappa ^1_{+}=\kappa ^1_{-}=1$, ${{\mathcal {S}}}=500$, $b_0=D_M=150$. $\kappa ^0_{-}=0.03,0.15,0.8$ and 2, respectively, for the cases A, B, C and D. The arrows denote the direction of wave propagation. The red and blue curves satisfy the monotonicity properties assigned to the points A, B, C and D (Color figure online)

**Fig. 6:**
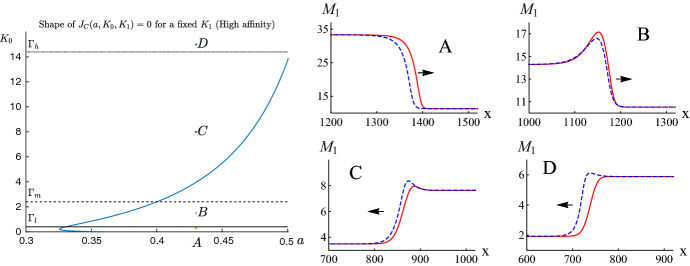
Left panel: The shape of the separatrix curve $J_C(a, K_0, K_1)=0$ for $c_b=0.2$ and a fixed $K_1=0.1$ in the $aK_0$ plane. Right panel: The profiles of the $M_1$ component of wave solutions corresponding to the following parameter pairs $(a, K_0)$: $A=(0.43,0.3)$, $B=(0.43,1.5)$, $C=(0.43,8)$, $D=(0.43,15)$ shown in the left panel. The red continuous curves correspond to traveling wave solutions of Eq. (25) generated by the initial condition $c(x,0)=0.2+H(x-1000)$ for $t=100$. The blue dashed curves correspond to traveling waves generated by system (5) by the same jump condition for *c* and the corresponding initial conditions for $M_1$ and $M_2$. Parameters used in the simulations: $D=300$, $L = 15$, $\kappa ^0_{+}=\kappa ^1_{+}=\kappa ^1_{-}=1$, ${{\mathcal {S}}}=500$, $b_0=D_M=150$. $\kappa ^0_{-}=0.03,0.15,0.8$ and 2, respectively, for the cases A, B, C and D. The arrows denote the direction of wave propagation. The red curves satisfy the monotonicity properties assigned to the points A, B, C and D (Color figure online)

For the parameter pair $(a, K_0)$ lying between the curves $\Gamma _l$ and $\Gamma _h$, the profile of the $M_1$-component of the corresponding wave solution is not monotonic, as illustrated in Figs. 5 and 6 .

Another feature of the profile of the $M_1$-component of wave solutions is that in the presence of two-site buffers, it may not be heteroclinic. Indeed, for the profile of the $M_1$ component to be heteroclinic, by (16), we must have that $M_1(c(-\infty )) \not = M_1(c(\infty ))$, and so that $M_1(c_b) \not = M_1(c_b+1)$. This motivates us to define the curve $\Gamma _m$ in the parameter $(a, K_0)$-plane as follows:

$$\begin{aligned} \Gamma _m{} & {} := \{(a, K_0):\, M_1(c_b) = M_1(c_b+1) \}. \\{} & {} \text {(the dashed (middle) horizontal line in Fig.}~5~ \text {and}~ 6) \end{aligned}$$

Therefore, for the parameter pair $(a, K_0)$ lying on the curve $\Gamma _m$, the profile of the $M_1$-component of the corresponding wave solution is homoclinic. Further, one can verify that for the parameter pair $(a, K_0)$ lying above $\Gamma _m$, the profile of the $M_1$-component of the corresponding wave solution is heteroclinic with $M_1(c(-\infty )) < M_1(c(\infty ))$, whereas the one lying below $\Gamma _m$, the corresponding profile of the $M_1$-component is heteroclinic with $M_1(c(-\infty )) > M_1(c(\infty ))$. The aforementioned discussions are illustrated in Figs. 5 and 6.

In contrast to the profile of the $M_1$ component of wave solutions, the profile of the $M_2$ component is always monotone increasing, as can be verified by (21) and (49). Further, by adding (18) and (21), we have that

$$\begin{aligned} \frac{d(M_1+M_2)}{\hbox {d}c} = \frac{b_0 K_0 \left( 1 + 2 K_1^{-1} c \right) }{\mathcal{K}(c)^2} > 0 \quad \hbox { on} \, \mathrm{I\!R}. \end{aligned}$$

Therefore, the profile of the total buffers in calcium-bound forms is always monotone increasing. Finally, let us emphasize that similar monotonicity/nonmonotonicity properties are shared by the profiles of traveling wave solutions to system (5), as it has been shown in Figs. 5 and 6 .

## Conclusions

In this work, we considered the properties of traveling wave solutions in systems of reaction-diffusion equations describing the dynamics of calcium ions in the presence of buffer molecules with two binding sites, which can reciprocally influence each other depending on their state, i.e., on whether they are free or occupied. Moreover, in the model we assumed that the binding takes place sequentially—the second after the first. Likewise the unbinding takes place in the inverse direction. It is obvious that, if the binding and unbinding processes at the two sites (in a given buffer molecule) are independent, then the action of ‘two-site’ buffers coincides with the action of two subpopulations of ‘one-site’ buffers with halved total densities. However, in general, one site and two site models give different quantitative as well as qualitative results. The proposed model with two site buffering molecules is described by system (5) (Buffered bistable system) or the corresponding system of odes for the traveling wave solutions (10). In Sects. 3.2–3.4, we derive from system (5), using the rapid buffering approximation, the Rapid buffered bistable system (23), (18), (21) and its parabolic counterpart (25), (18), (21). In Sect. 4, we formulate the condition for the existence of traveling waves (with positive speed) independently of how large is the buffer diffusion coefficient ($D_M$) and the total buffer concentration $b_0$. Basing on the asymptotic approximation of the proposed model, we can analyze the action of two-site buffers. In particular, we can compare it with the effect of one-site buffering molecules. In our study we chose the equilibrium level of cytosolic calcium ions (ground state) as $c_b=0.2$ ($\mu M$), but all the conclusions derived in the paper hold qualitatively for all positive values of $c_b$. First of all, as it was mentioned in Sect. 5.2.1, two-site buffers can stop calcium traveling waves for smaller values of the parameter $a \in (0,1/2)$, i.e., for higher excitability, with respect to one-site buffers (0.254 vs. 0.331). In the same subsection we analyze the shape of admissible regions in the $K_0K_1$-space for two chosen excitability parameters *a* equal to 0.38 (high excitability) and $a=0.43$ (low excitability). These shapes can have a very complicated form as it is seen from the middle and right panel of Fig. 3. In particular, there emerges an additional region guaranteeing the advancing wave propagation independently of the value $D_M b_0$.

In Sect. 5.2.3 we consider the problem of monotonicity of traveling wave profiles as solutions to the asymptotic equations (23), (18) and (21). It thus follows from relation (18) that for certain regions of parameter pairs $(K_0,K_1)$ the profiles of $M_1$ can be nonmonotone or even monotonically decreasing. Moreover, in Figs. 5, 6 we show numerically that the same monotonicity properties hold also for $M_1$ profiles of traveling wave solutions to the initial system (5). This consistency can be treated as a numerical proof of the validity of the asymptotic reduction.

Finally, two-site buffers can also facilitate the propagation of calcium traveling waves, i.e., induce their propagation, even in the case when it is impossible without their presence. This is shown numerically in Fig. 4 both for the rapid buffered bistable system and for the initial system (5) (*Buffered bistable system*).

This is a highly nonintuitive and unexpected result. Although it is well known that calcium buffers (including the major calcium buffers calmodulin and calretinin) generally have multiple binding sites that are not independent (Starovasnik et al. 1992; Schwaller 2010; Prins and Michalak 2011), the possible effects of this on the speed and existence of physiological waves remain almost entirely unexplored and unknown. Unfortunately, it is difficult to perform an experiment in which the interactions of the individual calcium buffer binding sites can be modulated while leaving the total amount of buffering unchanged; to our knowledge, this has never been attempted. Moreover, such an experiment would have to be performed ensuring that the buffering power remains within a relatively narrow region where the effects on waves of binding site dependence can be seen.

In future work, we intend to generalize our analysis to buffers in which the binding sites not only influence each other, but also have different binding and unbinding coefficients. This however will necessitate considering a system with more equations.

## Appendix: Existence of Traveling Waves for Sufficiently Fast Binding and Unbinding Reactions

In this appendix, we will consider the existence of solutions to the system

A.1 $$\begin{aligned} \begin{array}{l} D_c c'' - v c' + f(c) - \left[ k^0_+ b_0 c + \left( k^1_{+} c - k^0_{+} c - k^0_{-} \right) M_1 - \left( k^0_{+} c + k^1_{-} \right) M_2 \right] = 0, \\ D_{M} M''_1 - v M'_1 + k^0_+ b_0 c - \left( k^0_- + k^0_+ c + k^1_{+} c \right) M_1 + \left( k^1_{-} -k^0_+ c \right) M_2 = 0, \\ D_{M} M''_2 - v M'_2 + \left[ k^1_{+} M_1 c - k^1_- M_2 \right] = 0, \end{array} \end{aligned}$$

i.e., system (10). In the proof, we will use the modified method applied to a system describing one kind of buffer molecules with one binding site Kaźmierczak and Peradzynski (2011). As it was done in Sect. 3.1, let us rescale the kinetic coefficients of the considered system:

A.2 $$\begin{aligned} k^{0,1}_{\pm } =: L \kappa ^{0,1}_{\pm }. \end{aligned}$$

Hence, system (A.1) can be written in the form

A.3 $$\begin{aligned} \begin{array}{l} D_c c'' - v c' + f(c) - L \left[ \kappa ^0_+ b_0 c + \left( \kappa ^1_{+} c - \kappa ^0_{+} c - \kappa ^0_{-} \right) M_1 - \left( \kappa ^0_{+} c + \kappa ^1_{-} \right) M_2 \right] = 0, \\ D_{M} M''_1 - v M'_1 + L \left[ \kappa ^0_+ b_0 c - \left( \kappa ^0_- + \kappa ^0_+ c + \kappa ^1_{+} c \right) M_1 + \left( \kappa ^1_{-} -\kappa ^0_+ c \right) M_2 \right] = 0, \\ D_{M} M''_2 - v M'_2 + L \left[ \kappa ^1_{+} M_1 c - \kappa ^1_- M_2 \right] = 0. \end{array} \end{aligned}$$

In fact, we will assume that the parameter *L* is sufficiently large.

Let us define

A.4 $$\begin{aligned} \eta _2&= \left[ \kappa ^1_{+} M_1 c - \kappa ^1_- M_2 \right] \end{aligned}$$

A.5 $$\begin{aligned} \eta _1&= \kappa ^0_+ b_0 c - \left( \kappa ^0_- + \kappa ^0_+ c + \kappa ^1_{+} c \right) M_1 + \left( \kappa ^1_{-} -\kappa ^0_+ c \right) M_2. \end{aligned}$$

From (A.4) and (A.5) we obtain:

A.6 $$\begin{aligned} M_1(\xi )&= \dfrac{ b_0 \kappa ^0_+ \kappa ^1_- c(\xi ) + \kappa ^0_+ \eta _2(\xi ) c(\xi ) - \kappa ^1_- \eta _1(\xi ) - \kappa ^1_- \eta _2(\xi )}{\kappa ^0_+ \kappa ^1_+ c(\xi )^2+\kappa ^0_+ \kappa ^1_- c(\xi )+\kappa ^0_- \kappa ^1_-} \end{aligned}$$

A.7 $$\begin{aligned} M_2(\xi )&= \dfrac{b_0 \kappa ^0_+ \kappa ^1_+ c(\xi )^2-\kappa ^1_+ \eta _1(\xi ) c(\xi )-\kappa ^0_+\eta _2(\xi ) c(\xi )-\kappa ^1_+ \eta _2(\xi ) c(\xi )-\kappa ^0_- \eta _2(\xi ) }{ \kappa ^0_+ \kappa ^1_+ c(\xi )^2+\kappa ^0_+ \kappa ^1_- c(\xi )+\kappa ^0_- k^1_-}, \end{aligned}$$

and, in turn, the expressions for the derivatives of $M_1(\cdot )$ and $M_2(\cdot )$. Having the expressions for $M'_1(\cdot )$, $M'_2(\cdot )$, $M''_1(\cdot )$ and $M''_2(\cdot )$, we can write the second and third equation of system (A.1) in the form

A.8 $$\begin{aligned} D_M \dfrac{1}{d_M} A_{sec} \left( \begin{array}{c} \eta _1'' \\ \eta ''_2 \end{array} \right) - L \left( \begin{array}{c} \eta _1 \\ \eta _2 \end{array} \right) - \dfrac{D_M}{d_M^{\, 2}} \left( \begin{array}{c} \phi _1 \\ \phi _2 \end{array} \right) c''(\xi ) + \left( \begin{array}{c} {\tilde{\Phi }}_1\\ {\tilde{\Phi }}_2 \end{array} \right) = 0, \end{aligned}$$

where

A.9 $$\begin{aligned} d_M(c) := \kappa ^0_+ \kappa ^1_+ c(\xi )^2+\kappa ^0_+ \kappa ^1_- c(\xi )+\kappa ^0_- \kappa ^1_-, \nonumber \\ A_{sec} = \left( \begin{array}{ll} \kappa ^1_- &{}\quad - \kappa ^0_+ c(\xi ) + \kappa ^1_- \\ \kappa ^1_+ c(\xi ) &{}\quad \kappa ^0_- + \left( \kappa ^0_+ + \kappa ^1_+ \right) c(\xi ) \end{array} \right) , \end{aligned}$$

whereas ${\tilde{\Phi }}_1$ and ${\tilde{\Phi }}_2$ are functions of *c*, $c'$, $\eta _1$, $\eta _2$ and *v*. Finally

A.10 $$\begin{aligned} \phi _1= & {} b_0 \kappa ^0_+ \kappa ^1_- \left( \kappa ^0_- \kappa ^1_- -c^2 \kappa ^0_+ \kappa ^1_+ \right) \nonumber \\{} & {} + \, \kappa ^0_{+} \kappa ^1_{-} \left( \kappa ^1_{-} + 2 \kappa ^1_{+} c \right) \left( \eta _1 + \eta _2 \right) + \kappa ^0_{+} \left( \kappa ^1_{-} \kappa ^0_{-} - \kappa ^0_{+} \kappa ^1_{+} c^2 \right) \eta _2 \nonumber \\:= & {} \phi _{10} + \phi _{1}^\eta , \ \phi _2 = b_0 c \kappa ^0_+ (2 \kappa ^0_- +c \kappa ^0_+) \kappa ^1_- \kappa ^1_+ \nonumber \\{} & {} + \, \kappa ^0_+ \kappa ^1_+ c (2 \kappa ^0_{-} + \kappa ^0_+ c)\eta _2 +\kappa ^1_+ \left( -\kappa ^0_{-} \kappa ^1_{-} + \kappa ^0_+ \kappa ^1_+ c^2 \right) ( \eta _1 + \eta _2) := \phi _{20} + \phi _{2}^\eta .\nonumber \\ \end{aligned}$$

Let us note that

$$\begin{aligned} \dfrac{\phi _1(c)}{d_M(c)^2} = \theta _1(c), \quad \dfrac{\phi _2c)}{d_M(c)^2}= \theta _2(c) \end{aligned}$$

where $\theta _1$ and $\theta _2$ are defined in (18) and (21), respectively.

In (A.8) the functions ${\tilde{\Phi }}_1$, ${\tilde{\Phi }}_2$ contain the terms $v M'_1$ and $v M'_2$, hence the terms proportional to $c'(\cdot )$, $\eta '_1(\cdot )$ and $\eta '_2(\cdot )$ (obtained via the differentiation of (A.6) and (A.7)), as well as the products of these derivatives contained in the functions $D_M M''_1$ and $D_M M''_2$.

Next, let us note that $c''(\cdot )$ can be obtained by means of the first equation of system (A.1). This equation can be written as

$$\begin{aligned} c'' = L \dfrac{1}{D_c} \left( \eta _1 + 2 \eta _2 \right) + \dfrac{1}{D_c} \left( v c' - f(c) \right) := L \dfrac{1}{D_c} \left( \eta _1 + 2 \eta _2 \right) - \dfrac{1}{D_c} \Phi _c(c,c'). \end{aligned}$$

Equation (A.8) can thus be written as

A.11 $$\begin{aligned} D_M \dfrac{1}{d_M} A_{sec} \left( \begin{array}{c} \eta _1'' \\ \eta ''_2 \end{array} \right) - \chi \, L \left( \begin{array}{c} \phi _1 (\eta _1+ 2 \eta _2) \\ \phi _2 (\eta _1+2 \eta _2) \end{array} \right) - L \left( \begin{array}{c} \eta _1 \\ \eta _2 \end{array} \right) + \left( \begin{array}{c} {\tilde{\Phi }}_1 + \chi \, \phi _1 \Phi _c \\ {\tilde{\Phi }}_2 + \chi \, \phi _2 \Phi _c \end{array} \right) = 0,\nonumber \\ \end{aligned}$$

where

A.12 $$\begin{aligned} \chi =\dfrac{1}{d_M^{\, 2}} \dfrac{D_M}{D_c}=\dfrac{1}{d_M^{\, 2}} d. \end{aligned}$$

Consequently, we obtain the system:

A.13 $$\begin{aligned} D_M \left( \begin{array}{c} \eta _1'' \\ \eta ''_2 \end{array} \right) - L A_\textrm{sec}^* \left( \begin{array}{c} \eta _1 \\ \eta _2 \end{array} \right) - \chi (c) \, L A^*_\textrm{sec} \left( \begin{array}{c} \phi _1 (\eta _1+ 2 \eta _2) \\ \phi _2 (\eta _1+2 \eta _2) \end{array} \right) + \left( \begin{array}{c} \Phi _1 \\ \Phi _2 \end{array} \right) = 0,\nonumber \\ \end{aligned}$$

with

A.14 $$\begin{aligned} A^*_\textrm{sec} = \left( \begin{array}{ll} \kappa ^0_- + \left( \kappa ^0_+ + \kappa ^1_+ \right) c(\xi ) &{}\quad \kappa ^0_+ c(\xi ) - \kappa ^1_- \\ -\kappa ^1_+ c(\xi ) &{}\quad \kappa ^1_- \end{array} \right) , \end{aligned}$$

and

$$\begin{aligned} \left( \begin{array}{l} \Phi _1\\ \Phi _2 \end{array} \right) = A_\textrm{sec}^* \left( \begin{array}{c} {\tilde{\Phi }}_1 + \chi (c) \, \phi _1 \Phi _c \\ {\tilde{\Phi }}_2 + \chi (c) \, \phi _2 \Phi _c \end{array} \right) \,, \end{aligned}$$

hence the system

A.15 $$\begin{aligned} D_M \left( \begin{array}{c} \eta _1'' \\ \eta ''_2 \end{array} \right) - L A_\textrm{sec}^* \left( \begin{array}{ll} 1 + \chi \phi _1 &{}\quad 2 \chi (c) \phi _2 \\ \chi \phi _1 &{}\quad 1 + 2 \chi (c) \phi _2 \end{array} \right) \left( \begin{array}{c} \eta _1 \\ \eta _2 \end{array} \right) + \left( \begin{array}{c} \Phi _1 \\ \Phi _2 \end{array} \right) = 0, \end{aligned}$$

Let us consider the system corresponding to (A.8) for the variables $\eta :=\eta _1+\eta _2$, $\eta _2$. It is seen that this system has the form:

A.16 $$\begin{aligned} D_M \left( \begin{array}{c} \eta '' \\ \eta ''_2 \end{array} \right) - L A_d \left( \begin{array}{c} \eta \\ \eta _2 \end{array} \right) + \left( \begin{array}{l} \Phi \\ \Phi _2 \end{array} \right) = 0 \end{aligned}$$

where

A.17 $$\begin{aligned} A_d= & {} A_d(c,\eta ,\eta _2) \nonumber \\= & {} \left( \begin{array}{ll} \kappa ^0_- (1+ \chi \phi _1) +c \kappa ^0_+ (1+ \chi \phi _1 + \chi \phi _2) &{}\quad \kappa ^0_- (-1+\chi \phi _1) +c \kappa ^0_+\chi (\phi _1+\phi _2) \\ -\kappa ^1_+ c (1+\chi \phi _1) + \kappa ^1_- \chi \phi _2 &{}\quad \kappa ^1_- + c \kappa ^1_+ (1 -\chi \phi _1) + \kappa ^1_- \chi \phi _2 \end{array} \right) \,\nonumber \\ \end{aligned}$$

and

$$\begin{aligned} \Phi = \Phi _1 + \Phi _2 \end{aligned}$$

Let us note that the matrix $A_d$ is obtained from the matrix

$$\begin{aligned} K = A_\textrm{sec}^* \left( \begin{array}{ll} 1 + \chi \phi _1 &{}\quad 2 \chi \phi _2 \\ \chi \phi _1 &{}\quad 1 + 2 \chi \phi _2 \end{array} \right) \end{aligned}$$

via the relations

$$\begin{aligned}{} & {} A_{d11} = K_{11}+K_{21}, \quad A_{d12}=K_{12}-K_{21} +K_{22}-K_{11},\\{} & {} A_{d21}=K_{21}, \quad A_{d22}=K_{22}-K_{21}. \end{aligned}$$

We will first analyze the properties of the matrix

$$\begin{aligned} B:= A_d(c,\eta =0,\eta _2=0), \end{aligned}$$

where $\phi _{10}$ and $\phi _{20}$ are defined in (A.10).

This matrix can be written as

A.18 $$\begin{aligned} B= & {} \left( \begin{array}{ll} \kappa ^0_- +c \kappa ^0_+ &{}\quad -k^0_- \\ -c \kappa ^1_+ &{}\quad k^1_- + c k^1_+ \end{array} \right) \nonumber \\{} & {} + \chi \left( \begin{array}{ll} \kappa ^0_- \phi _{10} +c \kappa ^0_+ (\phi _{10} + \phi _{20}) &{}\quad \kappa ^0_- \phi _{10} +c k^0_+ (\phi _{10}+\phi _{20}) \\ - c \kappa ^1_+ \phi _{10} + \kappa ^1_- \phi _{20} &{}\quad - c \kappa ^1_+ \phi _{10} + \kappa ^1_- \phi _{20} \end{array} \right) \nonumber \\=: & {} A + \chi A_\chi (\phi _{10},\phi _{20}). \end{aligned}$$

Putting the expressions for $\phi _{10}$ and $\phi _{20}$ from (A.10) we obtain:

$$\begin{aligned} \begin{array}{l} A_{\chi 11} = A_{\chi 12} = b_0 \kappa ^0_{-} (\kappa ^0_{+})^2 \kappa ^1_{-}\left( \kappa ^0_{-} + c \left( ^0_{+} + c \kappa ^1_{+} \right) \right) \\ A_{\chi 21} = A_{\chi 22} = b_0 c \kappa ^0_{+} \kappa ^1_{-} \kappa ^1_{+} \left( -\kappa ^0_{-} \left( \kappa ^0_{+} - 2 \kappa ^1_{-} \right) + c \kappa ^0_{+} \left( \kappa ^1_{-} + c \kappa ^1_{+} \right) \right) . \end{array} \end{aligned}$$

### Lemma A.1

There exists a constant $c_b \in (0,1)$, such that for all sets of the coefficients $\kappa ^0_{\pm }>0$, $\kappa ^1_{\pm }>0$, the trace of the matrix *B* positive.

### Proof

As it follows from straightforward calculations, the trace of *B* is equal to:

A.19 $$\begin{aligned}{} & {} (\kappa ^0_{-} + \kappa ^1_{-} + c (\kappa ^0_{+} + \kappa ^1_{+})) \nonumber \\{} & {} \quad + b_0 \chi \kappa ^0_{+} \kappa ^1_{-} \left( \left( \kappa ^0_{-}\right) ^2 \kappa ^0_{+} + \kappa ^0_{-} c \left[ \left( \kappa ^0_{+} \right) ^2 + (-1 + c) \kappa ^0_{+} \kappa ^1_{+} + 2 \kappa ^1_{-} \kappa ^1_{+} \right] \right. \nonumber \\{} & {} \quad \left. + c^2 \kappa ^0_{+} \kappa ^1_{+} (\kappa ^1_{-} + c \kappa ^1_{+}) \right) \end{aligned}$$

The first component of the sum is obviously positive. To prove the positivity of the second component, let us note that the expression in the main bracket is positive for $\kappa ^0_{-} \ge 0$ and $c>0$, unless the coefficient $(\kappa ^0_{+})^2 + (-1 + c) \kappa ^0_{+} \kappa ^1_{+} + 2 \kappa ^1_{-} \kappa ^1_{+}$ is negative. Next, the minimal value of the second-order polynomial with respect to $\kappa ^0_{-}$ is equal to $-\Delta /(4 \kappa ^0_{+})$, where

$$\begin{aligned} -\Delta =4 c^2 (\kappa ^0_{+})^2 \kappa ^1_{+} (\kappa ^1_{-} + c \kappa ^1_{+}) - c^2\Big [ (\kappa ^0_{+})^2 + (-1 + c) \kappa ^0_{+} \kappa ^1_{+} + 2 \kappa ^1_{-} \kappa ^1_{+} \Big ]^2 . \end{aligned}$$

Suppose that $c<1$. Then, $(-1 + c) \kappa ^0_{+} \kappa ^1_{+} < 0$, but as we have assumed that $[ (\kappa ^0_{+})^2 + (-1 + c) \kappa ^0_{+} \kappa ^1_{+} + 2 \kappa ^1_{-} \kappa ^1_{+}]<0$, hence $[ (\kappa ^0_{+})^2 + (-1 + c) \kappa ^0_{+} \kappa ^1_{+} + 2 \kappa ^1_{-} \kappa ^1_{+}]^2 < [(-1 + c) \kappa ^0_{+} \kappa ^1_{+}]^2 = (-1 + c)^2 (\kappa ^0_{+})^2 (\kappa ^1_{+})^2$. It follows that

$$\begin{aligned}{} & {} (-\Delta ) > c^2 (\kappa ^0_+)^2 \Big [ 4 \kappa ^1_{+} \kappa ^1_{-} + 4 c (\kappa ^1_{+})^2 - (1 - 2 c +c^2) (\kappa ^1_{+})^2 \Big ] \\{} & {} \quad = 4 c^2 (\kappa ^0_+)^2 \kappa ^1_{+} \kappa ^1_{-} + c^2 (\kappa ^0_+)^2 (\kappa ^1_{+})^2 \Big [(-1 + 6 c - c^2) \Big ]. \end{aligned}$$

The second term of the above sum is positive for $c \in (0.1716, 5.8284)$ hence for $c \in (0.1716,1)$. If $c \ge 1$, then $[ (\kappa ^0_{+})^2 + (-1 + c) \kappa ^0_{+} \kappa ^1_{+} + 2 \kappa ^1_{-} \kappa ^1_{+}] >0$ and the second component in (A.19) is positive and growing with *c*. The lemma is proved. $\square$

### Lemma A.2

Let $c > c_b$, where $c_b$ is determined in Lemma A.1. Then, there exist ${{\mathcal {D}}}_1>0$ sufficiently small and $\mathcal{D}_2>{{\mathcal {D}}}_1$ sufficiently large, such that for $d b_0 \in [0,{{\mathcal {D}}}_1) \cup ({{\mathcal {D}}}_2,\infty )$, the eigenvalues of *B*, $\lambda _{10}$ and $\lambda _{20}$ are both positive.

### Proof

By straightforward calculation it follows that

A.20 $$\begin{aligned} Det(B) = d_M(c) \Big ( 1 + d \, (d_M(c))^{-2} b_0 \, \kappa ^0_{+}\kappa ^1_{-} (c^2 \kappa ^0_{+} \kappa ^1_{+} + \kappa ^0_{-} (\kappa ^0_{+} + 4 c \kappa ^1_{+})) \Big ) >0.\nonumber \\ \end{aligned}$$

As the eigenvalues of the matrix *B* are equal to

$$\begin{aligned} \begin{array}{l} \lambda _{10} = \dfrac{1}{2} \Big ( \hbox {Trace}(B) + \sqrt{\Big ( \hbox {Trace}(B)^2 - 4 \hbox {Det}(B) \Big )}, \\ \lambda _{20} = \dfrac{1}{2} \Big ( \hbox {Trace}(B) - \sqrt{\Big ( \hbox {Trace}(B)^2 - 4 \hbox {Det}(B) \Big )}, \end{array} \end{aligned}$$

then according to Lemma A.1, they are real and positive if only

A.21 $$\begin{aligned} \hbox {Trace}(B)^2 - 4 \hbox {Det}(B) > 0. \end{aligned}$$

For $d=0$, $\hbox {Trace}(B)^2 - 4 \hbox {Det}(B)=\hbox {Trace}(A)^2 - 4 \hbox {Det}(A)$ is equal to

$$\begin{aligned} (\kappa ^0_{-})^2 + \left( \kappa ^1_{-} + c \left( -\kappa ^0_{+} + \kappa ^1_{+} \right) \right) ^2 + 2 \kappa ^0_{-} \left( -\kappa ^1_{-}+ c \left( \kappa ^0_{+} + \kappa ^1_{+} \right) \right) . \end{aligned}$$

This expression is positive unless the coefficient $(-\kappa ^1_{-}+ c (\kappa ^0_{+} + \kappa ^1_{+}))$ is negative. Then, the minimal value of the quadratic polynomial with respect to $\kappa ^0_{-}$ is equal to

$$\begin{aligned} \begin{array}{l} \left( \kappa ^1_{-} + c \left( -\kappa ^0_{+} + \kappa ^1_{+} \right) \right) ^2 - \left( -\kappa ^1_{-}+ c \left( \kappa ^0_{+} + \kappa ^1_{+} \right) \right) ^2= 4 c \left( \kappa ^1_{-}-c \kappa ^0_{+} \right) \cdot \kappa ^1_{+}. \end{array} \end{aligned}$$

The last expression is positive, because $\kappa ^1_{-} -c \kappa ^0_{+}>c \kappa ^1_{+}>0$ for $c>0$. It follows that the eigenvalues of *A* are positive for some $d \in [0,d_1)$ for some $d_1>0$ sufficiently small.

Now, let us consider the case of $d\cdot b_0$ sufficiently large . Then, by (A.20), the *Det*(*B*) grows proportionally to $d b_0$, whereas $(Trace(B))^2$ grows proportionally to $(d b_0)^2$. It follows that condition (A.21) is satisfied, so the second part of the thesis of the lemma holds. The lemma is proved. $\square$

Now, we can generalize the result concerning the eigenvalues of the matrix *B* expressed in Lemma A.2, to the matrix $A_d$, which in contrast to the matrix *B*, is dependent on the functions $\eta$, $\eta _2$. In this case to retain the positivity of the eigenvalues of $A_d$, we must assume that the $C^0$ norms of the functions $\eta$ and $\eta _2$ are sufficiently small.

### Lemma A.3

Let $c > c_b$, where $c_b$ is determined in Lemma A.1. Suppose that $\Vert {\tilde{\eta }}(\cdot )\Vert _{C^3(\mathrm{I\!R})} < C$, $\Vert {\tilde{\eta }}_2(\cdot )\Vert _{C^3(\mathrm{I\!R})} < C$, $\Vert {\tilde{\eta }}(\cdot )\Vert _{C^0(\mathrm{I\!R})} < s_0$, $\Vert {\tilde{\eta }}_2(\cdot )\Vert _{C^0(\mathrm{I\!R})} < s_0$, with $s_0$ sufficiently small (but independent of the parameter *L*). Then, there exist ${{\mathcal {D}}}_1>0$ sufficiently small and ${{\mathcal {D}}}_2>{{\mathcal {D}}}_1$ sufficiently large, such that for $d b_0 \in [0,{{\mathcal {D}}}_1) \cup ({{\mathcal {D}}}_2,\infty )$, the eigenvalues of $A_d(c,{\tilde{\eta }},{\tilde{\eta }}_2)$ are both positive for all $\xi \in \mathrm{I\!R}$.

### Definition 1

Given the functions $c(\cdot )$, ${\tilde{\eta }}(\cdot )$ and ${\tilde{\eta }}_2(\cdot )$, let $\lambda _1(\xi )$ and $\lambda _2(\xi )$ denote the positive eigenvalues of the matrix $A_d(c,{\tilde{\eta }},{\tilde{\eta }}_2)$.

### Remark

For simplicity, in our denotation we have not emphasized in Definition 1 the fact that the eigenvalues depend on the functions $c(\cdot )$, ${\tilde{\eta }}(\cdot )$ and ${\tilde{\eta }}_2(\cdot )$. So in fact we should use the notation:

$$\begin{aligned} \lambda _1\left[ c,{\tilde{\eta }},{\tilde{\eta }}_2 \right] (\xi ), \ \ \lambda _2\left[ c,{\tilde{\eta }},{\tilde{\eta }}_2 \right] (\xi ). \end{aligned}$$

$$\square $$

Let us start from the analysis of the homogeneous system adjoint to the homogeneous version of system (A.16), i.e.,

A.22 $$\begin{aligned} D_M \left( \begin{array}{c} \eta '' \\ \eta ''_2 \end{array} \right) - L A_d(c,{\tilde{\eta }},{\tilde{\eta }}_2)^T(\xi ) \left( \begin{array}{c} \eta \\ \eta _2 \end{array} \right) = 0. \end{aligned}$$

We will show that Eq. (A.22) has no bounded smooth solutions in $C^2(\mathrm{I\!R})$ except for zero solutions.

Consider the matrix

A.23 $$\begin{aligned} S(\xi ) = \Big ( {{\mathcal {C}}}_1(\xi ) \, \, \mathcal{C}_2(\xi ) \Big ) \end{aligned}$$

where ${{\mathcal {C}}}_1$ and ${{\mathcal {C}}}_2$ are the column eigenvectors corresponding to the positive eigenvalues $\lambda _{1,2}(\xi )$ of the matrix $A_d(c,{\tilde{\eta }},{\tilde{\eta }}_2)(\xi )$, i.e.,

A.24 $$\begin{aligned} S^{-1}(\xi ) \, A_d(c,{\tilde{\eta }},{\tilde{\eta }}_2)(\xi ) \, S(\xi ) = \left( \begin{array}{ll} \lambda _1(\xi ) &{}\quad 0 \\ 0 &{}\quad \lambda _2(\xi ) \end{array} \right) . \end{aligned}$$

### Remark

As the eigenvalues of the matrix $A_d(c,{\tilde{\eta }},{\tilde{\eta }}_2)$, the entries of the matrix $S(\xi )$, i.e., depend on the functions *c*, ${\tilde{\eta }}$, and ${\tilde{\eta }}_2$. So,

$$\begin{aligned} S(\xi )=S \left[ c,{\tilde{\eta }},{\tilde{\eta }}_2 \right] (\xi ). \Box \end{aligned}$$

Let us write

A.25 $$\begin{aligned} S^{-1}(\xi ) = \left( \begin{array}{l} {{\mathcal {K}}}_1(\xi ) \\ {{\mathcal {K}}}_2(\xi ) \end{array} \right) , \end{aligned}$$

where ${{\mathcal {K}}}_1$ and ${{\mathcal {K}}}_2$ are row vectors such that ${{\mathcal {K}}}_i \cdot {{\mathcal {C}}}_j = \delta _{ij}$, $i,j=1,2$.

Simultaneously, the matrix $A_d(c,{\tilde{\eta }},{\tilde{\eta }}_2)(\xi )^T$ can be diagonalized by the matrix $S(\xi )^T$, i.e.,

$$\begin{aligned} S(\xi )^T \, A_d(c,{\tilde{\eta }},{\tilde{\eta }}_2)(\xi )^T \, [S^{-1}(\xi )]^T = \left( \begin{array}{cc} \lambda _1(\xi ) &{} 0 \\ 0 &{} \lambda _2(\xi ) \end{array} \right) . \end{aligned}$$

Let us denote

$$\begin{aligned}{}[S^{-1}(\xi )]^T =: J(\xi ). \end{aligned}$$

In fact, according to (A.26), we have

A.26 $$\begin{aligned}{}[S^{-1}(\xi )]^T = \Big ( {{\mathcal {K}}}^T_1(\xi ) \, \, \, {{\mathcal {K}}}^T_2(\xi ) \Big ). \end{aligned}$$

Then, $S(\xi )^T = \{ [S(\xi )^{-1}]^T \}^{-1}= J(\xi )^{-1}$. Multiplying Eq. (A.22) by $J^{-1}(\xi )$ we obtain the system

A.27 $$\begin{aligned} D_M J^{-1}(\xi ) J(\xi ) J(\xi )^{-1} \left( \begin{array}{c} \eta '' \\ \eta ''_2 \end{array} \right) {-} L \left( \begin{array}{ll} \lambda _1(\xi ) &{}\quad 0 \\ 0 &{}\quad \lambda _2(\xi ) \end{array} \right) \, J^{-1}(\xi ) \left( \begin{array}{c} \eta \\ \eta _2 \end{array} \right) = 0,\qquad \quad \end{aligned}$$

hence, by denoting

$$\begin{aligned} J(\xi )^{-1} \left( \begin{array}{c} \eta \\ \eta _2 \end{array} \right) = S(\xi )^T \left( \begin{array}{c} \eta \\ \eta _2 \end{array} \right) = \left( \begin{array}{c} {{\mathcal {J}}}_{11}(\xi ) \eta + {{\mathcal {J}}}_{12}(\xi ) \eta _2 \\ {{\mathcal {J}}}_{21}(\xi ) \eta + {{\mathcal {J}}}_{22}(\xi ) \eta _2 \end{array} \right) =: \left( \begin{array}{c} \zeta _1 \\ \zeta _2 \end{array} \right) , \end{aligned}$$

the system

A.28 $$\begin{aligned} D_M \left( \begin{array}{c} \zeta _1'' \\ \zeta ''_2 \end{array} \right) - L \left( \begin{array}{ll} \lambda _1(\xi ) &{}\quad 0 \\ 0 &{}\quad \lambda _2(\xi ) \end{array} \right) \left( \begin{array}{c} \zeta _1 \\ \zeta _2 \end{array} \right) + \left[ D_M {{\mathcal {J}}}^{-1}(\xi ) \left( \begin{array}{c} \eta ''(\xi ) \\ \eta ''_2(\xi ) \end{array} \right) - D_M \left( \begin{array}{c} \zeta ''_1(\xi ) \\ \zeta ''_2(\xi ) \end{array} \right) \right] = 0.\nonumber \\ \end{aligned}$$

The term in the square bracket depends on $c''(\xi ), \, c'(\xi ), \, \eta '(\xi ), \, \eta (\xi ), \, \eta '_2(\xi ), \eta (\xi )$ and $\eta _2(\xi )$. To be more precise

$$\begin{aligned} \left[ {{\mathcal {J}}}^{-1}(\xi ) \left( \begin{array}{c} \eta ''(\xi ) \\ \eta ''_2(\xi ) \end{array} \right) - \left( \begin{array}{c} \zeta ''_1(\xi ) \\ \zeta ''_2(\xi ) \end{array} \right) \right]= & {} {{\mathcal {J}}}^{-1}(\xi ) \left( \begin{array}{c} \eta ''(\xi ) \\ \eta ''_2(\xi ) \end{array} \right) - \left( {{\mathcal {J}}}^{-1}(\xi ) \left( \begin{array}{c} \eta (\xi ) \\ \eta _2(\xi ) \end{array} \right) \right) '' \\= & {} - 2 {{\mathcal {J}}}^{-1}(\xi )' \left( \begin{array}{c} \eta '(\xi ) \\ \eta '_2(\xi ) \end{array} \right) - {{\mathcal {J}}}^{-1}(\xi )'' \left( \begin{array}{c} \eta (\xi ) \\ \eta _2(\xi ) \end{array} \right) \\= & {} - 2 {{\mathcal {J}}}^{-1}(\xi )' \Big [ S(\xi ) \left( \begin{array}{c} \zeta _1(\xi ) \\ \zeta _2(\xi ) \end{array} \right) \Big ]' \\{} & {} - {{\mathcal {J}}}^{-1}(\xi )'' \left( \begin{array}{c} \eta (\xi ) \\ \eta _2(\xi ) \end{array} \right) \\= & {} - \Big [ 2 {{\mathcal {J}}}^{-1}(\xi )' \, {{\mathcal {J}}}'(\xi ) + \mathcal{J}^{-1}(\xi )'' \, {{\mathcal {J}}}(\xi ) \Big ] \left( \begin{array}{c} \zeta _1(\xi ) \\ \zeta _2(\xi ) \end{array} \right) \\{} & {} - 2 {{\mathcal {J}}}^{-1}(\xi )' \, S{{\mathcal {J}}}(\xi ) \left( \begin{array}{c} \zeta _1(\xi ) \\ \zeta _2(\xi ) \end{array} \right) ' \\=: & {} -Z \left( \begin{array}{c} \zeta _1(\xi ) \\ \zeta _2(\xi ) \end{array} \right) - Z_1 \left( \begin{array}{c} \zeta _1(\xi ) \\ \zeta _2(\xi ) \end{array} \right) ', \end{aligned}$$

where the matrices $Z(\xi )$ and $Z_1(\xi )$ do not depend on the parameter *L*. It follows that system (A.28) can be written in the form:

A.29 $$\begin{aligned} D_M \left( \begin{array}{c} \zeta _1'' \\ \zeta ''_2 \end{array} \right) - L \left( \begin{array}{ll} \lambda _1(\xi ) &{}\quad 0 \\ 0 &{} \quad \lambda _2(\xi ) \end{array} \right) \left( \begin{array}{c} \zeta _1 \\ \zeta _2 \end{array} \right) - D_M Z(\xi ) \left( \begin{array}{c} \zeta _1 \\ \zeta _2 \end{array} \right) - D_M Z_1(\xi ) \left( \begin{array}{c} \zeta _1 \\ \zeta _2 \end{array} \right) ' = 0.\nonumber \\ \end{aligned}$$

As system (A.29) is linear, then we can normalize it by the demand that one of the norms: $\Vert \zeta '_1\Vert _{C^2(\mathrm{I\!R})}$ or $\Vert \zeta '_2\Vert _{C^2(\mathrm{I\!R})}$ is equal to 1 and the other is not bigger than 1. To fix our attention, we will assume that

A.30 $$\begin{aligned} \Vert \zeta _1\Vert _{C^1(\mathrm{I\!R})} = 1 \quad \textrm{and} \quad \Vert \zeta _2\Vert _{C^1(\mathrm{I\!R})} \le 1. \end{aligned}$$

Let us denote

$$\begin{aligned} {\underline{\lambda }}_i = \min _{\xi \in \mathrm{I\!R}} \, \lambda _i(\xi ), \quad i=1,2. \end{aligned}$$

Suppose that the function $\zeta _1(\cdot )$ attains a positive maximum at a point $\xi ^* \in \mathrm{I\!R}$. Then, as *Z* and $Z_1$ do not depend on *L*, $\zeta '_1(\xi ^*)=0$ and $\zeta ''_1(\xi ^*)<0$, hence

A.31 $$\begin{aligned} \zeta _1(\xi ^*) (L \lambda _1(\xi ) - D_M z_{11}(\xi _*)) \le D_M (|Z_{12}(\xi _*)| |\zeta _2(\xi _*)|+ |Z_{112}(\xi _*)||\zeta '_2(\xi _*)|)\nonumber \\ \end{aligned}$$

thus, due to the assumed normalization

A.32 $$\begin{aligned}{} & {} \zeta _1(\xi _*) \le \frac{2}{L {\underline{\lambda }}_1}D_M (|Z_{12}(\xi _*)| + |Z_{112}(\xi _*)|) \nonumber \\{} & {} \quad \le \frac{C}{L{\underline{\lambda }}_1}. \end{aligned}$$

Likewise, if $\zeta _1$ attains a negative minimum at $\xi _*$, then

A.33 $$\begin{aligned} \zeta _1(\xi _*) \le \frac{2}{L{\underline{\lambda }}_1}D_M (|Z_{12}(\xi _*)| + |Z_{112}(\xi _*)|) \le \frac{C}{L{\underline{\lambda }}_1}, \end{aligned}$$

where *C* is independent of *L*.

In the same way, we conclude that

A.34 $$\begin{aligned} |\zeta _2(\xi )| \le \frac{C}{L {\underline{\lambda }}_2}. \end{aligned}$$

### Remark

In analyzing the extrema of the functions $\zeta _1$ and $\zeta _2$, we assumed that they are attained at finite values of $\xi$. The same reasoning can be applied also to extrema attained at infinities. Consider, e.g., a positive maximum of $\zeta _1$ achieved at $+\infty$. It can happen, if there exists a sequence of increasing maxima, so it can be analyzed as above. In the other, monotone case, for sufficiently large $\xi$ we must have $\zeta '_1(\xi )>0$, $\zeta '_1(\xi ) \rightarrow 0$ for $\xi \rightarrow \infty$, and $\zeta ''_1(\xi ) < 0$, so we can also use the arguments applied to the case of finite $\xi ^*$. The other cases can be analyzed similarly. $\square$

Next, it follows from the assumed normalization that the second derivatives of the functions $\zeta _1$ and $\zeta _2$ are bounded from above and below by numbers independent of *L*.

### Lemma A.4

Let $\Vert u''(L;\cdot )\Vert _{C^0(\mathrm{I\!R})} \le d_2$, where $d_2>0$ is independent of *L*. Suppose that as $L \rightarrow \infty$, $\Vert u(L;\cdot )\Vert _{C^0(\mathrm{I\!R}0)} \le \dfrac{d_0}{L}$ for some finite $d_0$ independent of *L*. Then, the function $u'(L;\cdot )$ satisfies the estimate $\Vert u'(L;\cdot )\Vert \le \dfrac{d_1}{L^{1/2}}$, where $d_1$ is a constant independent of *L*.

### Proof

Suppose that at some $\xi =\xi _0$ we have $u'(\xi _0) = L^{-p} d_1 > 0$ for some $d_2 >0$ and $p \ge 0$ as $L \rightarrow \infty$. Then, $u'(\xi ) > L^{-p} d_1/2$ on an interval $(\xi _0, \xi _0+L^{-p} \dfrac{d_1}{2d_2})$. Suppose next that $u(\xi _0) = c_1 L^{-1}$ for some $|c_1| \ge 0$ independent of *L*. Then, at $\xi =\xi _2:=\xi _0 + L^{-p} \dfrac{d_1}{2d_2}$ the value of *u* would be larger than $c^1 L^{-1} + L^{-2p} \dfrac{d^2_1}{4 d_2}$. It follows that, if $p<1/2$, then $L^{-2p}/L^{-1}$ tends to $\infty$ as $L \rightarrow \infty$, then $u(\xi _2)/L^{-1} \rightarrow \infty$ as $L \rightarrow \infty$ in contradiction with $\Vert u(L;\cdot )\Vert _{C^0(\mathrm{I\!R}0)} \le \dfrac{d_0}{L}$. Similar reasoning can be applied, when $u'(\xi _0) < 0$. In conclusion, $p \ge 1/2$ and the final estimate of the lemma holds. $\square$

According to Lemma A.4, we conclude that $|\zeta '_i(\xi )|<d_1 L^{-1/2}$, $i=1,2$, hence using inequalities (A.31), (A.33), (A.34), we conclude that

$$\begin{aligned} \Vert \zeta _1(\cdot )\Vert _{C^1(\mathrm{I\!R})} = \sup _{\xi \in \mathrm{I\!R}} \Big ( |\zeta _1(\xi )| + |\zeta '_1(\xi )| \Big ) \le \frac{C}{L{\underline{\lambda }}_1} + \dfrac{d_1}{L^{1/2}} < 1. \end{aligned}$$

As $d_1$ is independent of *L*, then for *L* sufficiently large we arrive at contradiction with the normalization condition (A.30). As the transformation $(\eta ,\eta _2) \mapsto (\zeta _1,\zeta _2)$ is smooth and invertible, then we have shown the validity of the following lemma.

### Lemma A.5

Suppose that $\Vert c(\cdot )\Vert _{C^2(\mathrm{I\!R})}<C$, $\Vert {\tilde{\eta }}(\cdot )\Vert _{C^0(\mathrm{I\!R})} < s_0$, $\Vert {\tilde{\eta }}_2(\cdot )\Vert _{C^0(\mathrm{I\!R})} < s_0$, with $s_0$ sufficiently small (but independent of the parameter *L*), $\Vert {\tilde{\eta }}(\cdot )\Vert _{C^2(\mathrm{I\!R})} \le C$ and $\Vert {\tilde{\eta }}_2(\cdot )\Vert _{C^2(\mathrm{I\!R})} \le C$. Then, for *L* sufficiently large, there is no $C^2$-bounded nonzero solution to system (A.29).

As the transformation $(\eta ,\eta _2) \mapsto (\zeta _1,\zeta _2)$ is smooth and invertible, then Lemma A.5 implies the following lemma.

### Lemma A.6

Consider the homogeneous system corresponding to Eq. (A.22):

A.35 $$\begin{aligned} D_M \left( \begin{array}{c} \eta '' \\ \eta ''_2 \end{array} \right) - L A_d(c,{\tilde{\eta }},{\tilde{\eta }}_2)^T \left( \begin{array}{c} \eta \\ \eta _2 \end{array} \right) = 0. \end{aligned}$$

Suppose that the functions $c(\cdot )$, ${\tilde{\eta }}$ and ${\tilde{\eta }}_2$ satisfy the conditions of Lemma A.5. Then, for *L* sufficiently large, there is no $C^2$-bounded nonzero solution to system (A.35).

Now, we will consider the system corresponding to system (A.16):

A.36 $$\begin{aligned} D_M \left( \begin{array}{c} \eta '' \\ \eta ''_2 \end{array} \right) - L A_d(c,{\tilde{\eta }},{\tilde{\eta }}_2)(\xi ) \left( \begin{array}{c} \eta \\ \eta _2 \end{array} \right) + \left( \begin{array}{l} \Phi (c,{\tilde{\eta }},{\tilde{\eta }}_2)(\xi ) \\ \Phi _2(c,{\tilde{\eta }},{\tilde{\eta }}_2)(\xi ) \end{array} \right) = 0 \end{aligned}$$

with the functions $c(\cdot )$, ${\tilde{\eta }}(\cdot )$ and ${\tilde{\eta }}_2$ fixed and satisfying conditions listed in Lemma A.5. Let us recall that, under these conditions, the matrix $A_d(c,{\tilde{\eta }},{\tilde{\eta }}_2)(\xi )$ can be diagonalized according to (A.24) by means of the matrix $S^{-1}(\xi )$ given by (A.26). Thus, multiplying system (A.36) by the matrix $S^{-1}(\xi )$, we obtain by means of (A.24) the system:

A.37 $$\begin{aligned} \left( \begin{array}{c} \zeta _1'' \\ \zeta ''_2 \end{array} \right) - \dfrac{L}{D_M} \left( \begin{array}{ll} \lambda _1(\xi ) &{}\quad 0 \\ 0 &{}\quad \lambda _2(\xi ) \end{array} \right) \left( \begin{array}{c} \zeta _1 \\ \zeta _2 \end{array} \right) - \Theta (\xi ) \left( \begin{array}{c} \zeta _1 \\ \zeta _2 \end{array} \right) - \Theta _1(\xi ) \left( \begin{array}{c} \zeta _1 \\ \zeta _2 \end{array} \right) ' + \left( \begin{array}{l} \Psi _1 \\ \Psi _2 \end{array} \right) = 0,\nonumber \\ \end{aligned}$$

where

$$\begin{aligned} \begin{array}{l} \left( \begin{array}{c} \Psi _1(\xi ) \\ \Psi _2(\xi ) \end{array} \right) = \dfrac{1}{D_M} S^{-1}(\xi ) \left( \begin{array}{l} \Phi (c,{\tilde{\eta }},{\tilde{\eta }}_2)(\xi ) \\ \Phi _2(c,{\tilde{\eta }},{\tilde{\eta }}_2)(\xi ) \end{array} \right) . \end{array} \end{aligned}$$

Let us note that the matrices $\Theta$ and $\Theta _1$ correspond to matrices *Z* and $Z_1$ defined after (A.28) by replacing ${{\mathcal {J}}}(\xi )$ by $S^{-1}(\xi )$. In this way, by denoting

$$\begin{aligned} {\tilde{L}}=\dfrac{L}{D_M}, \end{aligned}$$

system (A.37) can be written in the form:

A.38 $$\begin{aligned} \begin{array}{l} \left( \begin{array}{c} \zeta _1'' \\ \zeta ''_2 \end{array} \right) - {\tilde{L}} A_f (\xi ) \left( \begin{array}{c} \zeta _1 \\ \zeta _2 \end{array} \right) - \Theta _1(\xi ) \left( \begin{array}{c} \zeta _1 \\ \zeta _2 \end{array} \right) ' + \left( \begin{array}{l} \Psi _1(\xi ) \\ \Psi _2(\xi ) \end{array} \right) = 0, \end{array} \end{aligned}$$

where

$$\begin{aligned} A_f := \left( \begin{array}{ll} \lambda _1(\xi ) + \theta _{11}(\xi ) {\tilde{L}}^{-1} \ {} &{}\quad \theta _{12}(\xi ) {\tilde{L}}^{-1} \\ \theta _{21}(\xi ) {\tilde{L}}^{-1} \ {} &{}\quad \lambda _2(\xi ) + \theta _{22}(\xi ) {\tilde{L}}^{-1} \end{array} \right) , \end{aligned}$$

where $\theta _{ij}$ are the entries of the matrix $\Theta$.

### Lemma A.7

Let $Q=\{q_{ij}\}_{i,j=1,2}$ be a matrix independent of ${\tilde{L}}$. Let us consider the matrix

$$\begin{aligned} \left( \begin{array}{cccc} 0 &{}\quad 0 &{}\quad 1 &{}\quad 0 \\ 0 &{}\quad 0 &{}\quad 0 &{}\quad 1 \\ q_{11} + b_{11} &{}\quad q_{12} + b_{12} &{}\quad b_{13} &{}\quad b_{14} \\ q_{21} + b_{21}&{}\quad q_{22} + b_{22} &{}\quad b_{23} &{}\quad b_{24} \\ \end{array} \right) . \end{aligned}$$

where $b_{kl}$, $k=1,2$, $l=1,2,3,4$ behave like ${\tilde{L}}^{-1}$ as ${\tilde{L}} \rightarrow \infty$. Suppose that $Trace (Q)> 0$, $Det(Q)>0$ and $Trace(Q)^2 - 4 Det(Q)>0$. Then, for ${\tilde{L}} \rightarrow \infty$, the matrix has 4 real different eigenvalues, two positive and two negative, which can be expressed in the form:

$$\begin{aligned} \pm \dfrac{1}{\sqrt{2}} \sqrt{ \hbox {tr}(Q) \pm \sqrt{\hbox {tr}(Q)^2 - 4 \hbox {det}(Q)}} + O({\tilde{L}}^{-1}). \end{aligned}$$

### Proof

The proof follows from the implicit function theorem. $\square$

To proceed, let us note that system (A.38) can be written by introducing additional dependent variables corresponding to the first derivatives ($\nu +1:=\zeta '$ and $\nu _2:=\zeta '_2$) as a first order system (of four equations), i.e.,

A.39 $$\begin{aligned} \left( \begin{array}{l} \zeta '_1 \\ \zeta '_2 \\ \nu '_1 \\ \nu '_2 \end{array} \right) - {\tilde{L}} \left( \begin{array}{c} \begin{array}{cccc} 0 \, &{} 0 \, \, \, \, \,&{} \, \, \, \, \,1 &{} \,0 \\ 0 \, &{} 0 \, \, \, \, \, &{} \, \, \, \, \,0 &{} 1 \end{array} \\ \begin{array}{cc} A_f (\xi ) \, \, \, \, &{} \, \, \, \, \Theta _1(\xi ) \end{array} \end{array} \right) \left( \begin{array}{l} \zeta _1 \\ \zeta _2 \\ \nu _1 \\ \nu _2 \end{array} \right) = - \left( \begin{array}{c} 0 \\ 0 \\ \Psi _1(\xi ) \\ \Psi _2(\xi ) \end{array} \right) . \end{aligned}$$

System (A.39) can be formally treated as a linear system. For this representation of system (A.16), we can use Lemma 4.2 in Palmer (1984). According to Lemma A.7, the matrix multiplying ${\tilde{L}}$ for every $x \in \mathrm{I\!R}$, the $4 \times 4$ matrix at the left-hand side of the above system has two positive and two negative eigenvalues (as we assume that the functions ${\tilde{c}}$, ${\tilde{\eta }}$ and ${\tilde{\eta }}_2$ satisfy the conditions of Lemma A.5), so the dimensions of the stable and unstable subspaces are equal to 2. It follows that the left-hand side operator in (A.39) is Fredholm of index 0. Obviously, Lemma A.5 implies that the system adjoint to the homogeneous counterpart of system (A.39) has no nonzero solutions bounded in $C^1(\mathrm{I\!R})$ class. It follows that, for $A_f$ and $(\Psi _1,\Psi _2)^T$ treated as given functions, system (A.39) has a unique $C^1(\mathrm{I\!R})$ solution.

Consequently, given $A_f$, $\Psi _1$ and $\Psi _2$, system (A.38) has a unique $C^2$-bounded solution. Let us establish estimates for the derivatives of this solution. To do this we will make stronger assumptions concerning the functions ${\tilde{c}}$, ${\tilde{\eta }}$ and ${\tilde{\eta }}_2$. Namely, we will additionally assume that

A.40 $$\begin{aligned} \Vert {\tilde{c}} \Vert _{C^3(\mathrm{I\!R})}, \ \Vert {\tilde{\eta }} \Vert _{C^3(\mathrm{I\!R})} \ \ \text{ and } \ \ \Vert {\tilde{\eta }}_2 \Vert \Vert _{C^3(\mathrm{I\!R})} \quad \text{ are } \text{ bounded }. \end{aligned}$$

First, let us estimate first the $C^0$-norms of the solutions to system (A.38). Suppose that at $\xi =\xi _*$ the function $\zeta _1(\xi )$ attains a positive maximum. According to the Remark after inequality (A.34) we can confine ourselves to the case $\xi _* \in \mathrm{I\!R}$. It follows from the first equation of the system that $\zeta (\xi _*) \le C {\tilde{L}}^{-1}$. Proceeding in this way, we can prove that

$$\begin{aligned} \Vert \zeta _1(\cdot )\Vert _{C^0(\mathrm{I\!R})}\le {\tilde{C}} {\tilde{L}}^{-1}, \quad \Vert \zeta _2(\cdot )\Vert _{C^0(\mathrm{I\!R})} \le {\tilde{C}} {\tilde{L}}^{-1}. \end{aligned}$$

Moreover, according to Lemma A.5 the above estimates should be written as

A.41 $$\begin{aligned} \Vert \zeta _1(\cdot )\Vert _{C^0(\mathrm{I\!R})}\le C {\tilde{L}}^{-1} \Vert \Psi _1 \Vert _{C^0(\mathrm{I\!R})}, \quad \Vert \zeta _2(\cdot )\Vert _{C^0(\mathrm{I\!R})} \le C {\tilde{L}}^{-1} \Vert \Psi _1 \Vert _{C^0(\mathrm{I\!R})}. \end{aligned}$$

Next by differentiation of the equations in system (A.38) with respect to $\xi$, and using the estimates of $C^0$-norms, we can conclude that

A.42 $$\begin{aligned} \Vert \zeta '_1(\cdot )\Vert _{C^0(\mathrm{I\!R})} \le C {\tilde{L}}^{-1} \Vert \Psi _1 \Vert _{C^1(\mathrm{I\!R})}, \quad \Vert \zeta '_2(\cdot )\Vert _{C^0(\mathrm{I\!R})} \le C {\tilde{L}}^{-1} \Vert \Psi _2 \Vert _{C^1(\mathrm{I\!R})}. \end{aligned}$$

By means of the above estimates, it follows from Eq. (A.38) that $\zeta ''_1(\xi )$ and $\zeta ''_2(\xi )$ can be bounded from above and below by a constant independent of ${\tilde{L}}$. Consequently, it follows from the differentiation of Eq. (A.38) with respect to $\xi$ that

A.43 $$\begin{aligned} \zeta '''_1(\xi ) \quad \textrm{and} \quad \zeta '''_2(\xi ) \quad \text{ are } \text{ bounded } \text{ uniformly } \text{ for } \text{ all }~ {\xi \in \mathrm{I\!R}.} \end{aligned}$$

### Remark

The differentiation of the equations of system (A.38) is well determined as the entries of the matrix $\Theta$ is of $C^1$ class and the entries of the matrix $\Theta _1$ are of $C^2$ class, as we assume (see A.40) that the functions ${\tilde{c}}$, ${\tilde{\eta }}$ and ${\tilde{\eta }}_2$ are of $C^3$ class. Next, the eigenvalues $\lambda _1$ and $\lambda _2$ depend only on these functions (but not of its derivatives) and $\Psi _1$ and $\Psi _2$ are of $C^2$ class. $\square$

In view of the fact that the transformation $(\eta ,\eta _2) \mapsto (\zeta _1,\zeta _2)$ acting from $C^2(\mathrm{I\!R})$ to $C^2(\mathrm{I\!R})$ is well defined and invertible, then, according to Lemma A.5, the following lemma holds.

### Lemma A.8

System (A.35) has no nonzero $C^2(\mathrm{I\!R})$-bounded solutions $(\eta (\cdot ),\eta _2(\cdot ))$. System (A.36) has a unique $C^2$-bounded solution.

### Proof

The second claim of the lemma can be proved by repeating the reasoning given after system (A.39). $\square$

Next, due to the properties of the transformation $(\eta ,\eta _2) \mapsto (\zeta _1,\zeta _2)$, it follows that according to (A.41) and (A.42) we can write

A.44 $$\begin{aligned} \Vert \eta (\cdot )\Vert _{C^1(\mathrm{I\!R})} \le C {\tilde{L}}^{-1} \Vert \Psi _1 \Vert _{C^1(\mathrm{I\!R})}, \quad \Vert \eta _2(\cdot )\Vert _{C^1(\mathrm{I\!R})} \le C {\tilde{L}}^{-1} \Vert \Psi _2 \Vert _{C^1(\mathrm{I\!R})}. \end{aligned}$$

Differentiating Eq. (A.16) twice with respect to $\xi$ we obtain

A.45 $$\begin{aligned} D_M \left( \begin{array}{c} \eta '''' \\ \eta ''''_2 \end{array} \right) - L A_d(\xi ) \left( \begin{array}{c} \eta '' \\ \eta ''_2 \end{array} \right)= & {} L \left[ 2 A_d(\xi )' \left( \begin{array}{l} \eta ' \\ \eta '_2 \end{array} \right) + A_d(\xi )'' \left( \begin{array}{l} \eta \\ \eta _2 \end{array} \right) \right] \nonumber \\{} & {} - \left( \begin{array}{l} \Phi ''(\xi ) \\ \Phi ''_2(\xi ) \end{array} \right) =: \left( \begin{array}{l} \widetilde{{{\mathcal {T}}}}(\xi ) \\ \widetilde{{{\mathcal {T}}}}_2(\xi ) \end{array} \right) \end{aligned}$$

Using (A.44), we conclude that the right-hand side of the above equation can be estimated by a constant not depending on *L* for all $\xi \in \mathrm{I\!R}$. Multiplying the both sides of the last equation by $S^{-1}(\xi )$ we obtain the equation:

A.46 $$\begin{aligned} \begin{array}{l} D_M S^{-1}(\xi ) \left( \begin{array}{c} \eta '''' \\ \eta ''''_2 \end{array} \right) - L \left( \begin{array}{ll} \lambda _1(\xi ) &{} 0 \\ 0 &{} \lambda _2(\xi ) \end{array} \right) \left( S^{-1}(\xi ) \left( \begin{array}{c} \eta '' \\ \eta ''_2 \end{array} \right) \right) = S^{-1}(\xi ) \left( \begin{array}{l} \widetilde{{{\mathcal {T}}}}(\xi ) \\ \widetilde{{{\mathcal {T}}}}_2(\xi ) \end{array} \right) \end{array}\nonumber \\ \end{aligned}$$

As

$$\begin{aligned} S^{-1}(\xi ) \left( \begin{array}{c} \eta '''' \\ \eta ''''_2 \end{array} \right) = \Big [ S^{-1}(\xi ) \left( \begin{array}{c} \eta ''\\ \eta ''_2 \end{array} \right) \Big ]'' - 2 S^{-1}(\xi )' \left( \begin{array}{c} \eta '''\\ \eta '''_2 \end{array} \right) - S^{-1}(\xi )'' \left( \begin{array}{c} \eta ''\\ \eta ''_2 \end{array} \right) . \end{aligned}$$

In view of the first remark after (A.42), the second and the third derivatives of the functions $\eta$ and $\eta _2$ are of the order of *O*(1), hence by denoting

$$\begin{aligned} \left( \begin{array}{l} \vartheta _1 \\ \vartheta _2 \end{array} \right) := S^{-1}(\xi ) \left( \begin{array}{c} \eta '' \\ \eta ''_2 \end{array} \right) \end{aligned}$$

We can write Eq. (A.46) as:

A.47 $$\begin{aligned} \begin{array}{l} D_M \left( \left( \begin{array}{l} \vartheta _1 \\ \vartheta _2 \end{array} \right) \right) '' - L \left( \begin{array}{ll} \lambda _1(\xi ) &{}\quad 0 \\ 0 &{}\quad \lambda _2(\xi ) \end{array} \right) \left( \begin{array}{l} \vartheta _1 \\ \vartheta _2 \end{array} \right) = \left( \begin{array}{l} {{\mathcal {T}}}(\xi ) \\ {{\mathcal {T}}}_2(\xi ) \end{array} \right) , \end{array} \end{aligned}$$

where the functions ${{\mathcal {T}}}$ and ${{\mathcal {T}}}_2$ are of the order of *O*(1) and independent of *L*. It thus follows from the maximum principle and the Remark after (A.34) that

$$\begin{aligned} \Vert \eta ''\Vert _{C^0(\mathrm{I\!R})} \le L^{-1} O(1), \quad \Vert \eta ''_2\Vert _{C^0(\mathrm{I\!R})} \le L^{-1} O(1). \end{aligned}$$

Next, using the $C^0(\mathrm{I\!R})$ boundedness of the functions $\eta '''$ and $\eta '''_2$ and using the arguments from the proof of Lemma A.4, we can show that

$$\begin{aligned} \Vert \eta '''\Vert _{C^0(\mathrm{I\!R})} \le {L}^{-1/2} \, O(1), \quad \Vert \eta '''_2\Vert _{C^0(\mathrm{I\!R})} \le {L}^{-1/2} \, O(1). \end{aligned}$$

We have thus proved the following lemma.

### Lemma A.9

Suppose that $\eta$, $\eta _2$ denote a solution to Eq. (A.16), i.e.,

A.48 $$\begin{aligned} D_M \left( \begin{array}{c} \eta '' \\ \eta ''_2 \end{array} \right) - L A_d \left( \begin{array}{c} \eta \\ \eta _2 \end{array} \right) + \left( \begin{array}{l} \Phi \\ \Phi _2 \end{array} \right) = 0. \end{aligned}$$

Suppose that the solution satisfies the estimate $\Vert \eta (\cdot )\Vert _{C^2(\mathrm{I\!R})} \le C$, $\Vert \eta _2(\cdot )\Vert _{C^2(\mathrm{I\!R})} \le C$, where the constant *C* does not depend on *L*. Then, the following estimates hold:

$$\begin{aligned} \begin{array}{l} \Vert \eta (\cdot )\Vert _{C^2(\mathrm{I\!R})} + \Vert \eta _2(\cdot )\Vert _{C^2(\mathrm{I\!R})}\le L^{-1} \, {{\mathcal {C}}} \Big (\Vert c(\cdot )\Vert _{C^3(\mathrm{I\!R})} + \Vert \Phi [\eta ,\eta _2,c](\cdot ) \Vert _{C^3(\mathrm{I\!R})} + \Vert \Phi [\eta ,\eta _2,c](\cdot ) \Vert _{C^3(\mathrm{I\!R})} \Big ), \\ \Vert \eta '''\cdot )\Vert _{C^0(\mathrm{I\!R})} + \Vert \eta '''_2(\cdot )\Vert _{C^0(\mathrm{I\!R})} \le L^{-1/2} \, {{\mathcal {C}}} \Big (\Vert c(\cdot )\Vert _{C^3(\mathrm{I\!R})} + \Vert \Phi [\eta ,\eta _2,c](\cdot ) \Vert _{C^3(\mathrm{I\!R})} + \Vert \Phi [\eta ,\eta _2,c](\cdot ) \Vert _{C^3(\mathrm{I\!R})} \Big ), \end{array} \end{aligned}$$

where the constant ${{\mathcal {C}}}$ depends on the $C^2$ properties of the entries of the matrix $A_d$, but does not depend on *L* as $L \rightarrow \infty$.

Let us note that the first equation of system (A.1) can be written as

$$\begin{aligned} D_c c'' - v c' + f(c) - L \eta _1 - 2 L \eta _2 = 0 \end{aligned}$$

and consequently in the form independent of *L*:

$$\begin{aligned} D_c c'' - v c' + f(c) + D_M ( M''_1 + 2 M''_2) - v (M'_1+2 M'_2) = 0. \end{aligned}$$

Differentiating (A.6) and (A.7) we can subsequently write it in the form:

A.49 $$\begin{aligned}{} & {} \Big ( D_c + D_M ({\widetilde{\phi }}(c,\eta ) \Big )) c'' +2 D_M \Big ( \theta '(c) + \nu _1(\eta _1,\eta _2,c) \Big ) \, (c')^2 \nonumber \\{} & {} \quad - v \Big ( 1+\theta (c) + \nu _0(\eta _1,\eta _2,c) \Big ) c' + f(c) + \Phi _c(c,c',\eta ,\eta ',\eta '',v) = 0, \end{aligned}$$

where

$$\begin{aligned} {\widetilde{\phi }}(c,\eta ):= (\phi _1 + 2 \phi _2)(d_M)^{-2} \end{aligned}$$

and $\phi _1$ and $\phi _2$ are defined in (A.10), whereas $d_M$ in (A.9). As it follows from (24) and (A.10),

A.50 $$\begin{aligned} {\widetilde{\phi }}(c,\eta ) = \theta (c) + \nu _2(c,\eta _1,\eta _2), \end{aligned}$$

where $\nu _2$ is a linear function of $\eta _1$ and $\eta _2$, vanishing for $\eta _1=\eta _2=0$. Its form can be deduced from (A.10) to (A.9). Exactly, the same properties have the functions $\nu _1$ and $\nu _0$. Moreover, as it is easy to check that similar properties are shared by the function $\Phi _c$ with respect to $\eta _1$, $\eta _2$ as well as with respect to their derivatives.

Our aim is to prove the existence of a traveling wave solution to system (A.1) for sufficiently large values of the parameter *L* and to show that as $L \rightarrow \infty$ this traveling wave solution tends together with its speed of propagation to the heteroclinic pair given by the asymptotic Eq. (23). Our tool will be the implicit function theorem in Banach spaces of differentiable functions.

### Definition 2

For $=i=0,1,2$, let $B_i$ denote the sub-space of functions *u*(*z*) belonging to $BC^i(\mathrm{I\!R})$ and tending to finite limits as $z \rightarrow \pm \infty$ together with their derivatives (which tend to zero). Let $B_{i0}$ denote the subspace of $B_i$ consisting of functions *u* satisfying the condition:

A.51 $$\begin{aligned} u(0) = \frac{1}{2} [u(-\infty )+u(\infty )]. \end{aligned}$$

The norms in the spaces $B_j$ are taken to be

$$\begin{aligned} \Vert u \Vert _{B_j} = \sum _{k=0}^j \sup _{z \in \mathrm{I\!R}^1} \left| \frac{\hbox {d}^k}{\hbox {d} z^k} u(z) \right| . \end{aligned}$$

In these norms $B_i$ and $B_{i0}$ are Banach spaces. $\square$

We are now in a position to formulate our existence problem for system (A.1). According to what was said above, upon defining

A.52 $$\begin{aligned} \lambda ^2 = L^{-1}, \end{aligned}$$

and upon the diffeomorphic change of variables:

A.53 $$\begin{aligned} (\eta _1,\eta _2) \mapsto (\eta ,\eta _2) := (\eta _1+\eta _2,\eta _2), \end{aligned}$$

we can rewrite the system in the following form:

A.54 $$\begin{aligned} (Q(c,\eta ,\eta _2,v,\lambda ), P(c,\eta ,\eta _2,v,\lambda )) =( 0, 0), \end{aligned}$$

where *Q* is given by the left-hand side of (A.49) with $\eta _1$ replaced by $\eta - \eta _2$ and

$$\begin{aligned} P = \left( \begin{array}{l} \eta \\ \eta _2 \end{array} \right) - {{\mathcal {R}}}_d(c,\eta ,\eta _2,\lambda ) \left( \begin{array}{l} \Phi (c,\eta ,\eta _2,v) \\ \Phi _2(c,\eta ,\eta _2,v) \end{array} \right) , \end{aligned}$$

where we explicitly denoted the dependence of the functions $\Phi$ and $\Phi _2$ on the speed parameter *v*. Here, given the set $\{\kappa ^0_-,\kappa ^0_+,\kappa ^1_-,\kappa ^1_+\}$, the corresponding values of the parameters $db_0$ and $c_b$ (according to Lemma A.2), $v \in \mathrm{I\!R}$ and $\lambda ^2 \ll 1$, the functions $c, \eta ,\eta _2 \in C^3(\mathrm{I\!R})$ in the matrix $A_d$ and the source terms $\Phi$ and $\Phi _2$, ${{\mathcal {R}}}_d(c,\eta ,\eta _2,\lambda ) (\Phi ,\Phi _2)$ is the unique bounded over the whole line solution to system (A.16). Thus, solutions to system (A.1) are defined as the simultaneous zeros of the mappings *Q* and *P*.

Let us note that for $\lambda = 0$ the quadruple $(c,\eta ,\eta _2,v)=(C,0,0,v_0)$, where $(C,v_0)$ is a heteroclinic pair for Eq. (23) provided implicitly by Lemma 1. This follows from the fact that for $\lambda = 0$, $\eta \equiv 0$, $\eta _2 \equiv 0$, the terms $\nu _j$, $j=0,1,2$ and $\Phi _c$ vanish identically.

### Lemma A.10

The following statements hold:

(1): *Q* is a well-defined mapping from some open neighborhood of the point $(c,\eta ,\eta _2,v,\lambda )=(C,0,0,v_0,0)$ in the space $B_{30} \times B_3 \times B_3 \times \mathrm{I\!R}\times \mathrm{I\!R}$ to the space $B_1$.
(2): $$Q(C,0,0,v_0,0) = 0$$.
(3): For all $\lambda$ sufficiently close to 0, *Q* treated as a function of $(c,\eta ,\eta _2,v)$ is continuously Frechet differentiable at the asymptotic solution $(C,0,0,v_0)$.

### Proof

The proof of the lemma follows by a straightforward checking. $\square$

We have, by using (A.50),

A.55 $$\begin{aligned}{} & {} DQ|_{\lambda =0}[\delta c, \delta \eta , \delta \eta _2, \delta v] \nonumber \\{} & {} \quad =\Big ( D_c + D_M \theta (C) \Big ) \delta c'' + \Big ( 2 D_M \theta '(C) C - v_0 (1+ \theta (C) ) \Big ) \, \delta c' \nonumber \\{} & {} \quad \quad + \dfrac{\partial }{\partial c} \Big [ D_M \theta (c) C'' + 2 D_M \theta '(c) C'^2 - v_0 \theta (c) C' + f'(c) \Big ] \Big |_{c=C} \, \delta c - \delta v ( 1+\theta (C)) C' \nonumber \\{} & {} \quad \quad + \dfrac{\partial }{\partial \eta } \Big ( D_M \nu _2(C,\eta ,\eta _2) C'' + 2 D_M \nu _1(C,\eta ,\eta _2) C'^2 - v_0 \nu _0(C,\eta ,\eta _2) C' \Big ) \Big |_{\eta =0,\eta _2=0} \delta \eta \nonumber \\{} & {} \quad \quad + \dfrac{\partial }{\partial \eta _2} \Big ( D_M \nu _2(C,\eta ,\eta _2) C'' + 2 D_M \nu _1(C,\eta ,\eta _2) C'^2 - v_0 \nu _0(C,\eta ,\eta _2) C' \Big ) \Big |_{\eta =0,\eta _2=0} \delta \eta _2 \nonumber \\{} & {} \quad \quad + \sum _{(j)=0,1,2} \dfrac{\partial }{\partial \eta ^{(j)}} \Phi _c(C,C',\eta ,\eta _2,\eta ',\eta '_2,\eta '',\eta ''_2,v) \, \delta \eta ^{(j)} \nonumber \\{} & {} \quad \sum _{(j)=0,1,2} \dfrac{\partial }{\partial \eta ^{(j)}_2} \Phi _c(C,C',\eta ,\eta _2,\eta ',\eta '_2,\eta '',\eta ''_2,v) \, \delta \eta ^{(j)}_2 \nonumber \\{} & {} \quad = \Big \{ \Big ( D_c + D_M \theta (C) \Big ) \delta c'' + D_M \Big ( 2 \theta '(C) C - v_0 (1+ \theta (C) ) \Big ) \, \delta c' \nonumber \\{} & {} \quad \quad + \dfrac{\partial }{\partial c} \Big [ D_M \theta (c) C'' + 2 D_M \theta '(c) C'^2 - v_0 \theta (c) C' + f'(c) \Big ] \Big |_{c=C} \, \delta c - \delta v ( 1+\theta (C)) C' \Big \} \nonumber \\{} & {} \quad \quad + {{\mathcal {F}}}([\delta \eta , \delta \eta _2]) := L_0 \delta c - \delta v (1+ \theta (C)) C' + {{\mathcal {F}}}([\delta \eta , \delta \eta _2]). \end{aligned}$$

### Remark

For simplicity, we are using the same symbols for the functions $\nu _2$, $\nu _1$ and $\nu _0$ expressed in terms of $\eta$ and $\eta _1$ instead of $\eta _1$ and $\eta _2$. $\square$

### Lemma A.11

Suppose that the parameter $db_0$ satisfies the assumptions of Lemma A.2. The following statements hold:

(1): For any $\lambda \ne 0$, with $| \lambda |$ sufficiently small, $P(\cdot ,\cdot ,\cdot ,\cdot ,\lambda )$ is a well-defined mapping from some open neighborhood ${{\mathcal {N}}}_P$ of the point $(c,\eta ,\eta _2,v)=(C,0,0,v_0)$ in the space $B_{30} \times B_3 \times B_3 \times \mathrm{I\!R}$ to the space $B_3$.
(2): For every $(c,\eta ,\eta _2,v) \in {{\mathcal {N}}}_P$, $\Vert P(c,\eta ,\eta _2,v,\lambda ) \Vert _{B_3} \rightarrow (\eta ,\eta _2)^T$ for $\lambda \rightarrow 0$.
(3): For any $\lambda \ne 0$, with $| \lambda |$ sufficiently small, is continuously Frechet differentiable close to the asymptotic solution $(C,0,0,v_0)$. Moreover, $DP|_\lambda [\delta c, \delta \eta , \delta \eta _2] \rightarrow (\eta ,\eta _2)^T$ as $\lambda \rightarrow 0$.

### Proof

Points 1. and 2. follow from Lemmata A.8 and A.9. To prove point 3., it suffices to show that as $\lambda \rightarrow 0$, i.e., $L \rightarrow \infty$,

$$\begin{aligned} \begin{array}{l} {{\mathcal {R}}}_d(c+\delta c,\eta +\delta \eta ,\eta _2 + \delta \eta _2,\lambda ) \left( \begin{array}{l} \Phi ([c + \delta c,\eta + \delta \eta ,\eta _2 + \delta \eta _2]) \\ \Phi _2([c + \delta c,\eta + \delta \eta ,\eta _2 + \delta \eta _2]) \end{array} \right) \\ \quad -{{\mathcal {R}}}_d(c,\eta ,\eta _2,\lambda ) \left( \begin{array}{l} \Phi ([c,\eta ,\eta _2]) \\ \Phi _2([c,\eta ,\eta _2]) \end{array} \right) \underset{\lambda \rightarrow 0}{ \longrightarrow }\ 0. \end{array} \end{aligned}$$

This statement is true, because for $\delta c$, $\delta \eta$ and $\delta \eta _2$ sufficiently small in $C^2(\mathrm{I\!R})$-norm, the assumptions of Lemmata A.8 and  A.9 are fulfilled. $\square$

We have shown the validity of the following statement.

### Lemma A.12

The mapping *P* can be continuously extended to the value $\lambda =0$ by assuming

$$\begin{aligned} P(c,\eta ,\eta _2,v,0) \equiv 0. \end{aligned}$$

Similarly, $DP|_\lambda$ can be continuously extended to the value $\lambda =0$ by assuming

$$\begin{aligned} DP|_\lambda \, [\delta c, \delta \eta , \delta \eta _2] = 0. \end{aligned}$$

In view of Lemmata A.10–A.12, we can use the implicit function theorem to prove the existence of traveling waves for system (A.1). It thus suffices to show that the linear system

A.56 $$\begin{aligned} \begin{array}{l} DQ|_{\lambda =0}[\delta c, \delta \eta , \delta \eta _2,\delta v] = h_c \in B_1 \\ \delta \eta = h_\eta \in B_3 \\ \delta \eta _2 = h_{\eta _2} \in B_3 \end{array} \end{aligned}$$

defines an isomorphism between the spaces $B_{30} \times B_3 \times B_3 \times \mathrm{I\!R}$, i.e., (via Theorem 4.2-H in Taylor (1958)) and that the above system is boundedly invertible. Replacing $\delta \eta$ by $h_\eta$ and $\delta \eta _2$ by $h_{\eta _2}$ in the first equation of the above system becomes

A.57 $$\begin{aligned} L_0 \delta c - \delta v (1+ \theta (C)) C' = {\tilde{h}}_c, \end{aligned}$$

where

$$\begin{aligned} {\tilde{h}}_c = h_c - {{\mathcal {F}}}([h_\eta ,h_{\eta _2}]) \in B_1. \end{aligned}$$

Let us recall that according to Lemma 1, $w'(\xi )>0$ for all $\xi \in \mathrm{I\!R}$, so due the monotonicity of transformation (27), the profile $C(\cdot )$ of the traveling front to Eq. (A.49) with $\eta \equiv 0$, $\eta _2 \equiv 0$ (and $\lambda =0$), is monotonically increasing, i.e., $C'(\xi )>0$. Moreover, $C'(\xi ) \rightarrow 0$ as $\xi \rightarrow \pm \infty$. It follows that the homogeneous version of Eq. (A.57) has a unique bounded solution (up to a translation and a multiplicative constant) equal to $C'(\cdot )$, because the other linearly independent solution diverges at infinities. However, this function does not belong to the space $B_{30}$, because it does not satisfy condition (A.51). It may be easily shown that a unique (up to a multiplicative constant) solution to a conjugated equation has the form

$$\begin{aligned} C'(\xi ) \exp (\int _0^\xi a(s) ds ), \quad a(s) =\Big ( 2 D_M \theta '(C) C - v_0 (1+ \theta (C) ) \Big ), \end{aligned}$$

hence the condition of orthogonality to the right-hand side of the equation

A.58 $$\begin{aligned} L_0 \delta c = - \delta v (1+ \theta (C)) C' + {\tilde{h}}_c \end{aligned}$$

takes the form

$$\begin{aligned} \int _{\mathrm{I\!R}} C'(z) \exp \left( \int _0^z a(s) \hbox {d}s \right) \Big \{ - C'(z) (1+\theta (C(z)) \delta v - {\tilde{h}}_c(z) \Big \} dz = 0. \end{aligned}$$

In view of the fact that $C'(\xi )>0$ for all $\xi \in \mathrm{I\!R}$, the last equation is uniquely solvable with respect to $\delta v$.

*Leading order approximations*

As the functions $\Phi$ and $\Phi _2$ are independent of the parameter *L*, then, according to Lemma A.11 in the first nonzero approximations:

A.59 $$\begin{aligned} \begin{array}{l} \Vert \eta \Vert _{C^1(\mathrm{I\!R})} \approx L^{-1} O\Big ( \Vert \Phi [C,v](\cdot )\Vert _{C^1(\mathrm{I\!R})}+\Vert \Phi _2[C,v](\cdot )\Vert _{C^1(\mathrm{I\!R})} \Big ), \\ \Vert \eta _2 \Vert _{C^1(\mathrm{I\!R})} \approx L^{-1} O\Big ( \Vert \Phi [C,v](\cdot )\Vert _{C^1(\mathrm{I\!R})}+\Vert \Phi _2[C,v](\cdot )\Vert _{C^1(\mathrm{I\!R})} \Big ), \\ \Vert \eta \Vert _{C^2(\mathrm{I\!R})} \approx L^{-1} O\Big ( \Vert \Phi [C,v](\cdot )\Vert _{C^2(\mathrm{I\!R})}+\Vert \Phi _2[C,v](\cdot )\Vert _{C^2(\mathrm{I\!R})} \Big ), \\ \Vert \eta _2 \Vert _{C^2(\mathrm{I\!R})} \approx L^{-1} O\Big ( \Vert \Phi [C,v](\cdot )\Vert _{C^2(\mathrm{I\!R})}+\Vert \Phi _2[C,v](\cdot )\Vert _{C^2(\mathrm{I\!R})} \Big ), \end{array} \end{aligned}$$

and

A.60 $$\begin{aligned} \begin{array}{l} \Vert \eta \Vert _{C^3(\mathrm{I\!R})} \approx L^{-1/2} O\Big ( \Vert \Phi [C,v](\cdot )\Vert _{C^2(\mathrm{I\!R})}+\Vert \Phi _2[C,v](\cdot )\Vert _{C^2(\mathrm{I\!R})} \Big ), \\ \Vert \eta _2 \Vert _{C^3(\mathrm{I\!R})} \approx L^{-1/2} O\Big ( \Vert \Phi [C,v](\cdot )\Vert _{C^2(\mathrm{I\!R})}+\Vert \Phi _2[C,v](\cdot )\Vert _{C^2(\mathrm{I\!R})} \Big ) \end{array} \end{aligned}$$

Due to (A.55) we thus have

$$\begin{aligned} \Vert {\tilde{h}}_c \Vert _{C^0(\mathrm{I\!R})} \approx L^{-1} O(\Vert C\Vert _{C^2(\mathrm{I\!R})}), \end{aligned}$$

thus the principal change in the speed

$$\begin{aligned} \delta v = (v-v_0) \approx L^{-1} O(\Vert C\Vert _{C^2(\mathrm{I\!R})}). \end{aligned}$$

Consequently, using (A.57), we conclude that similarly

A.61 $$\begin{aligned} \Vert c-C\Vert _{C^2(\mathrm{I\!R})} \approx L^{-1} O(\Vert C\Vert _{C^2(\mathrm{I\!R})}) \end{aligned}$$

and

A.62 $$\begin{aligned} \Vert c-C\Vert _{C^3(\mathrm{I\!R})} \approx L^{-1/2} O(\Vert C\Vert _{C^3(\mathrm{I\!R})}) \end{aligned}$$

Finally, if we denote by $M_{10}$ and $M_{20}$ the asymptotic limit of (A.6), (A.7), that is:

A.63 $$\begin{aligned} M_{10}(\xi ) = M_{10}(C(\xi )) := \dfrac{\kappa ^0_+ b_0 C(\xi )}{\kappa ^0_- + \kappa ^0_+ C(\xi ) + \kappa ^0_+ \dfrac{\kappa ^1_{+}}{\kappa ^1_-} C^2(\xi )} \end{aligned}$$

and

A.64 $$\begin{aligned} M_{20}(\xi ) = M_{20}(C(\xi )):= \dfrac{\kappa ^1_+}{\kappa ^1_-} M_{10}(C(\xi )) C(\xi ). \end{aligned}$$

Let us denote

$$\begin{aligned}{} & {} M_{1-} = M_{10}(c_b), \quad M_{1+} = M_{10}(1+c_b), \\{} & {} M_{2-} =c_b \dfrac{\kappa ^1_+}{\kappa ^1_-} M_{1-}, \quad M_{2+} = (1+c_b) \dfrac{\kappa ^1_+}{\kappa ^1_-} M_{1+} \,. \end{aligned}$$

We have thus proved the following theorem.

### Theorem A.1

Suppose that $|\lambda | = L^{-1/2}$ and that the parameter $\mathcal{D}=db_0$ satisfies the conditions of Lemma A.2. Then, for every sufficiently small $|\lambda |$ sufficiently small, there exists a heteroclinic quadruple $\Big ( c(\cdot ;|\lambda |),M_{1}(\cdot ;|\lambda |),M_2(\cdot ,L),v(|\lambda |) \Big ) \in B_{30} \times B_{3} \times B_{3} \times \mathrm{I\!R}$ to system (A.1), such that $c(\pm \infty ,|\lambda |) = C(\pm \infty )$, $M_1(\pm \infty )=M_{1 \pm }$, $M_2(\pm \infty )=M_{2 \pm }$ and such that, as $\lambda \rightarrow 0$,

$$\begin{aligned} \begin{array}{l} \Vert c(\cdot ;|\lambda |)-C(\cdot )\Vert _{C^2(\mathrm{I\!R})} = O(\lambda ^2), \quad |v-v_0| = O(\lambda ^2), \\ \Vert M_1(\cdot ;|\lambda |)-M_{10}(\cdot )\Vert _{C^2(\mathrm{I\!R})} = O(\lambda ^2), \quad \Vert M_2(\cdot ;|\lambda |)- M_{20}(\cdot )\Vert _{C^2(\mathrm{I\!R})} = O(\lambda ^2), \end{array} \end{aligned}$$

and

$$\begin{aligned} \begin{array}{l} \Vert c(\cdot ;|\lambda |)-C(\cdot )\Vert _{C^3(\mathrm{I\!R})} = O(|\lambda |), \\ \Vert M_1(\cdot ;|\lambda |)-M_{10}(\cdot )\Vert _{C^3(\mathrm{I\!R})} = O(|\lambda |), \Vert M_2(\cdot ;|\lambda |)- M_{20}(\cdot )\Vert _{C^3(\mathrm{I\!R})} = O(|\lambda |). \end{array} \end{aligned}$$

## References

[CR1] Chen Y, Matveev V (2021). Stationary C$$\text{a}^{2+}$$ nanodomains in the presence of buffers with two binding sites. Biophys J.

[CR2] Clapham DE (2007). Calcium signaling. Cell.

[CR3] Dupont G, Falcke M, Kirk V, Sneyd J (2016). Models of calcium signalling, interdisciplinary applied mathematics.

[CR4] Falcke M (2004–2005) Reading the patterns in living cells - the physics of $${\rm Ca^{2+}}$$ signaling. Adv Phys 53:255–440

[CR5] Fitzhugh R (1960). Thresholds and plateaus in the Hodgkin-Huxley nerve conduction equations. J Gen Physiol.

[CR6] Fitzhugh R (1961). Impulses and physiological states in models of nerve membrane. Biophys J.

[CR7] Kaźmierczak B, Peradzynski Z (2011). Calcium waves with fast buffers and mechanical effects. J Math Biol.

[CR8] Kaźmierczak B, Sneyd J (2021). Speed of traveling waves for monotone reaction-diffusion systems as a function of diffusion coefficients. Phys D Nonlinear Phenom.

[CR9] Kaźmierczak B, Volpert V (2008). Calcium waves in systems with immobile buffers as a limit of waves for systems with nonzero diffusion. Nonlinearity.

[CR10] Kaźmierczak B, Volpert V (2008). Travelling calcium waves in systems with non-diffusing buffers. Math Models Methods Appl Sci.

[CR11] Keener J, Sneyd J (1998). Mathematical physiology.

[CR12] Matveev V (2018). Extension of rapid buffering approximation to $${\rm Ca^{2+}}$$ buffers with two binding sites. Biophys J.

[CR13] Nagumo J, Arimoto S, Yoshizawa S (1962). An active pulse transmission line simulating nerve axon. Proc IRE.

[CR14] Palmer KJ (1984). Exponential dichotomies and transversal homoclinic points. J Diff Equ.

[CR15] Prins D, Michalak M (2011). Organellar calcium buffers. Cold Spring Harb Perspect Biol.

[CR16] Schwaller B (2010). Cytosolic $$\text{ Ca}^{2+}$$ buffers. Cold Spring Harb Perspect Biol.

[CR17] Smith GD, Pearson JE, Keizer J, Fall CP, Marland ES, Wagner JM, Tyson JJ (2002). Modeling intracellular calcium waves and sparks. Computatiional cell biology.

[CR18] Sneyd J, Dale PD, Duffy A (1998). Traveling waves in buffered systems: applications to calcium waves. SIAM J Appl Math.

[CR19] Sorensen BR, Shea MA (1996). Calcium binding decreases the stokes radius of calmodulin and mutants R74A, R9OA, and R9OG. Biophys J.

[CR20] Starovasnik MA, Klevit RE, Su D-R, Beckingham K (1992). A series of point mutations reveal interactions between the calcium-binding sites of calmodulin. Protein Sci.

[CR21] Taylor AE (1958). Introduction to functional analysis.

[CR22] Tsai J-C (2007). Asymptotic stability of traveling wave fronts in the buffered bistable system. SIAM J Math Anal.

[CR23] Tsai J-C (2013). Do calcium buffers always slow down the propagation of calcium waves?. J Math Biol.

[CR24] Tsai J-C, Sneyd J (2005). Existence and stability of traveling waves in buffered systems. SIAM J Appl Math.

[CR25] Tsai J-C, Sneyd J (2011). Traveling waves in the buffered Fitzhugh-Nagumo model. SIAM J Appl Math.

[CR26] Volpert AI, Volpert VA, Volpert VA (1994). Traveling-wave solutions of parabolic systems.

[CR27] Wagner J, Keizer J (1994). Effects of rapid buffers on $${\rm Ca^{2+}}$$ diffusion and $${\rm Ca^{2+}}$$ oscillations. Biophys J.

